# SarA plays a predominant role in controlling the production of extracellular proteases in the diverse clinical isolates of *Staphylococcus aureus* LAC and UAMS-1

**DOI:** 10.1080/21505594.2020.1855923

**Published:** 2020-12-14

**Authors:** Aura M. Ramirez, Karen E. Beenken, Stephanie D. Byrum, Alan J. Tackett, Lindsey N. Shaw, Brittney D. Gimza, Mark S. Smeltzer

**Affiliations:** a Department of Microbiology and Immunology, University of Arkansas for Medical Sciences, Little Rock, AR, USA; b Department of Biochemistry and Molecular Biology, University of Arkansas for Medical Sciences, and Arkansas Children’s Research Institute, Little Rock, AR, USA; c Department of Cell Biology, Microbiology, and Molecular Biology, University of South Florida, Tampa, FL

**Keywords:** *Staphylococcus aureus*, extracellular protease, regulation, biofilm, *sarA*, *rot*, *mgrA*, *sarS*, *sarZ*, *sarR*

## Abstract

Using DNA affinity chromatography we demonstrate that the *S. aureus* regulatory proteins MgrA, Rot, SarA, and SarS bind DNA baits derived from the promoter regions associated with the genes encoding aureolysin, ScpAB, SspABC, and SplA-F. Three of four baits also bound SarR and SarZ, the exception in both cases being the ScpAB-associated bait. Using the USA300, methicillin-resistant strain LAC and the USA200, methicillin-sensitive strain UAMS-1, we generated mutations in the genes encoding each of these proteins alone and in combination with *sarA* and examined the impact on protease production, the accumulation of high molecular weight proteins, and biofilm formation. These studies confirmed that multiple regulatory loci are involved in limiting protease production to a degree that impacts all of these phenotypes, but also demonstrate that *sarA* plays a predominant role in this regard. Using *sarA* mutants unable to produce individual proteases alone and in combination with each other, we also demonstrate that the increased production of aureolysin and ScpA is particularly important in defining the biofilm-deficient phenotype of LAC and UAMS-1 *sarA* mutants, while aureolysin alone plays a key role in defining the reduced accumulation of alpha toxin and overall cytotoxicity as assessed using both osteoblasts and osteoclasts.

## Introduction

*Staphylococcus aureus* has capacity to cause a wide variety of diseases including acute toxemias, skin and soft tissue infections, sepsis, and deep secondary infections of essentially any tissue including bone [1]. This can be attributed to the arsenal of virulence factors *S. aureus* has at its disposal coupled with a complex and highly interactive regulatory circuit that allows for precise tailoring of this arsenal to meet the demands of different microenvironments within the host. Included among its many virulence factors are at least 15 extracellular proteases [2]. Five of these are variants of the exfoliative toxins (ETA, ETB, ETC, ETD, and ETE), which are serine proteases that exhibit exquisite specificity for desmoglein 1, a desmosomal cadherin responsible for maintaining the integrity of cell-to-cell adhesive structures in the skin [3]. Although the exfoliative toxins reportedly exhibit superantigen activity, this remains controversial, and it is clear that this highly specific proteolytic activity is the primary factor that defines the association between these proteases and the skin infections scalded skin syndrome and bullous impetigo [4,5]. The exfoliative toxins exhibit further specificity with respect to host species, and collectively they are only produced by approximately 5% of *S. aureus* strains [3,5].

Of the remaining 10 extracellular proteases, six are “serine protease-like” proteins encoded within the *spl* operon [6].These proteases (SplA, SplB, SplC, SplD, SplE, and SplF) also exhibit relatively limited substrate specificity and are not produced by all *S. aureus* strains [7–10]. For instance, a PCR-based survey of 167 *S. aureus* strains isolated from various forms of infection found that the *spl* operon is absent in approximately 16% of strains, and the entire operon is present in only 31% [10]. Among other strains, the presence of individual *spl* genes was found to range from 45% (*splD*) to 85% (*splF*). Nevertheless, the *spl*-encoded proteases have been implicated in the pathogenesis of pneumonia [8], and the highest total abundance of the six *spl* genes was observed in isolates obtained from pneumonia patients [10]. A recent report also found that mutations in *splD* and *splF* were associated with reduced fitness in a murine osteomyelitis model [11].

The other four extracellular proteases are the zinc metalloprotease aureolysin, the cysteine proteases ScpA and SspB, and the serine protease SspA. The genes encoding these proteases (*aur, scpA, sspB*, and *sspA*, respectively) are present in at least 98% of all *S. aureus* strains [10] and are arranged in three transcriptional units, with one containing only *aur*, the second containing *scpA* and the gene for its inhibitor staphostatin B (*scpB*), and the third containing *sspA, sspB*, and *sspC*, the latter encoding the inhibitor staphostatin C (*sspC*) [12–14]. With the exception of ScpA, fully active forms of these proteases are produced as part of a cascade, with aureolysin being required for full activation of SspA, and SspA being required in turn for full activation of SspB [14]. However, this activation cascade is not absolute in that proteolytically active forms of SspA and SspB are produced in an aureolysin mutant. By comparison to the exfoliative toxins and SplA-F, these proteases exhibit broad substrate specificity, and it is these proteases that have been studied most extensively as virulence factors [2,14]. These studies confirmed that these proteases degrade a variety of host proteins and serve important roles in pathogenesis by promoting tissue invasion, nutrient acquisition, and subversion of host defenses [15–25].

Based on such observations, it would be anticipated that eliminating the ability of *S. aureus* to produce these proteases would result in decreased virulence, but we and others have demonstrated that doing so in the USA300 strain LAC increases rather than decrease virulence in diverse forms of infection including sepsis and osteomyelitis [26–28]. Similarly, it would be anticipated that increased production of these proteases would increase virulence, but we have also demonstrated that mutation of the staphylococcal accessory regulator (*sarA*) results in the increased production of all of these proteases but reduced virulence in these same animal models [27–29]. These paradoxical observations can be explained by the fact that eliminating protease production results in the increased abundance of *S. aureus* surface-associated and extracellular virulence factors [26], while the increased production of proteases in *sarA* mutants results in the decreased accumulation of these virulence factors [27–29].

This suggests that, while it is important that *S. aureus* have the capacity to produce extracellular proteases, it is equally important that it has the capacity to limit the production of these proteases such that they serve their intended purposes on behalf of the bacterium without compromising the availability of its own virulence factors. *S. aureus* employs a complex and highly interactive regulatory circuit to ensure the availability of its virulence factors in amounts appropriate for different microenvironments within the host, and this includes extracellular proteases [30,31]. Two of the central elements in this regulatory circuit are the accessory gene regulator (*agr)*, which enhances the production of many extracellular proteins including proteases, and the staphylococcal accessory regulator (*sarA*), which functions as a repressor of protease production [14,32]. The regulatory functions of these two loci appear to be universal with respect to the genes/operons encoding individual proteases, including the exfoliative toxins [33], with *agr* activating and *sarA* repressing their production. However, there is some contradictory evidence in this regard. For instance, several reports concluded that mutation of *sarA* has no impact on transcription of the *spl* operon [6,14,34] but we have demonstrated that all six Spl proteases are present in increased amounts in conditioned media (CM) from stationary phase cultures of a LAC *sarA* mutant by comparison to CM from LAC itself [27–29]. Irrespective of this discrepancy, it is clear that mutation of *agr* results in a decrease in overall protease activity to a degree that can be correlated with an increased capacity to form a biofilm, while mutation of *sarA* results in an increase in overall protease activity to a degree that can be correlated with a decreased capacity to form a biofilm [32]. Although *sarA* enhances transcription of *agr* [35] this demonstrates that the regulatory impact of *sarA* on protease production and biofilm formation is independent of its interaction with *agr*. The phenotype of an *agr/sarA* double mutant also mimics that of an isogenic *sarA* mutant with respect to both of these phenotypes, demonstrating that the impact of mutating *sarA* is epistatic to that of mutating *agr* [32].

It has also become increasingly evident that the regulatory circuit modulating protease production extends far beyond these two loci. Indeed, mutations in *argR2, arlRS, atlR, codY, hisR, mgrA, mntR, msaABCR, nsaR, rbf, rex, rot, rpiRB, saeRS, sigB, sarR, sarS, sarT, sarU, sarV, sarX, sarZ, xdrA, yjbh*, and a number of uncharacterized putative regulatory loci have all been shown to impact the production of extracellular proteases [14,34,36–54]. A recent report directly compared the impact of mutating these loci on transcription of each of the four transcriptional units (*aur, scpAB, sspAB*, and *splA-F*) that encode aureolysin, ScpA, SspA, SspB, and SplA-F and concluded that the regulatory proteins encoded by seven of these loci (CodY, MgrA, Rot, SaeR, SarR, SarS, and SarA) constitute a primary network while those encoded by an additional seven loci (ArgR1, AtlR, MntR, Rbf, Rex, SarU, and XdrA) constitute a secondary network that functions primarily through its impact on regulatory elements within this primary network [34]. However, the impact of each of these loci with respect to individual protease genes/operons was found to vary widely. For instance, mutation of *codY* resulted in increased expression of the genes/operons encoding all of these proteases. This was also true of a *sarA* mutant with the exception of the *spl* operon, with expression levels of this operon being essentially unchanged in a *sarA* mutant [34]. As noted above, this is consistent with other reports examining the impact of *sarA* on *spl* transcription [6,14], but contradicted by our proteomics studies demonstrating that these proteases are present in increased amounts in CM from a LAC *sarA* mutant [27–29]. In contrast, mutation of *mgrA* was associated with decreased expression of all protease genes/operons other than that encoding *scpA*, which was largely unaffected in an *mgrA* mutant [34]. Mutation of the other four genes in the putative primary network resulted in increased expression of some protease genes/operons and decreased expression of others.

These observations emphasize the complexity of *S. aureus* regulatory circuits that modulate the production of extracellular proteases, and this implies that doing so is important in allowing *S. aureus* to employ post-translational tailoring of its virulence factor repertoire in a fashion that best meets its needs in different host microenvironments. Thus, it is important to better understand critical elements within the regulatory network that modulates protease production and the manner by which they do so. To this end, we employed an unbiased DNA-based protein capture approach to identify *S. aureus* regulatory proteins that bind to each of the promoter regions associated with *aur, scpAB, sspABC*, and the *splA-F* operon. Focusing on those regulatory loci that encode proteins that were captured by at least three of these DNA baits, we then generated mutations in the corresponding genes both alone and in combination with *sarA* and examined the impact on transcription of each gene/operon, overall protease activity, accumulation of alpha toxin and extracellular protein A (eSpa), and biofilm formation. We also investigated the role of individual proteases alone and in combination with each other on the accumulation of alpha toxin, biofilm formation, and cytotoxicity for osteoblasts and osteoclasts. To account for potential strain-dependent differences, all of these studies were done using the USA300, CC8, methicillin-resistant strain LAC and the USA200, CC30, methicillin-sensitive strain UAMS-1.

## Results

### *Identification of* S. aureus *regulatory proteins that bind protease-associated promoter regions*

**Figure 1.**
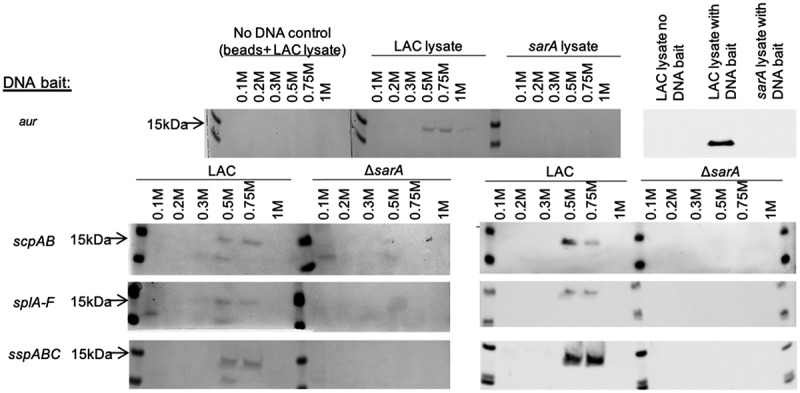
**Examination of captured proteins by SDS-PAGE and Western blot**. The region extending ~400 bp upstream of the genes and/or operons encoding extracellular proteases indicated on the left were used as DNA baits to capture proteins from whole cell lysates prepared from the USA300 strain LAC or its isogenic *sarA* mutant (Δ*sarA*). Proteins were eluted with increasing concentrations of salt and examined by SDS-PAGE (left side of each panel) and Western blot using an anti-SarA antibody (right side of each panel). Control samples included experiments done with a LAC lysate and no DNA ait (top)

We used an unbiased DNA-based protein capture approach to identify *S. aureus* regulatory proteins that bind to the promoter regions of each of the four genes/operons that encode aureolysin, ScpA, SspA and SspB, and the SplA-F proteases. Specifically, the DNA regions extending ~400 bp upstream of the translational start site of each of these genes/operons was amplified by PCR using 5ʹ biotinylated primers. The amplification products were mixed with whole cell lysates obtained from LAC or its isogenic *sarA* mutant. The mixtures were then incubated with streptavidin and loaded onto a magnetic column to isolate each DNA bait and its bound proteins. Bound proteins were dissociated from each bait and visualized by SDS-PAGE. All four DNA baits captured a 15 kDa protein (Figure 1). This protein was eluted at salt concentrations of ≥0.5 M, suggesting equivalent and relatively high-affinity binding to all baits. SarA is a 15 kDa protein, and this protein was not captured with any of the four DNA baits using lysates prepared from a LAC *sarA* mutant. These observations suggest this protein is likely to be SarA, which was confirmed by Western blot analysis using an anti-SarA antibody (Figure 1).

SarA has been shown to bind to the *aur* and *sspABC* promoter regions [49,52] but to our knowledge this is the first report demonstrating it also binds the *scpA* and *splA-F* promoters. The fact that SarA was captured using the *spl* bait is particularly significant in light of the discrepancy between reports suggesting that mutation of *sarA* does not impact expression of the *spl* operon [34] and our demonstration that these proteases, as well as aureolysin, ScpA, SspA, and SspB are present in increased amounts in conditioned medium (CM) from stationary phase cultures of a LAC *sarA* mutant by comparison to CM from LAC itself [27–29]. In addition, we previously used pCM11 [55] to generate transcriptional reporters in which DNA fragments upstream of each protease-encoding gene/operon were used to drive expression of the superfolder green fluorescent protein (sGFP), and when these were introduced into the parent strains and their isogenic *sarA* mutants, fluorescence was significantly higher in the *sarA* mutants with all four reporters including the *spl::gfp* reporter, and this was true in both LAC and UAMS-1 [56]. This confirms that SarA represses expression of all four of the transcriptional units encoding aureolysin, ScpA, SspA/SspB, and SplA-F, and the results of our binding assays suggest that it does so by directly binding to *cis* elements associated with all four of the relevant promoter regions.

Two other proteins, both of which were smaller than SarA, were also visible in some SDS-PAGE gels (Figure 1). One of these was eluted at low salt concentration (0.1 M) suggesting low affinity and potentially nonspecific binding. The other was a 12–13 kDa protein that was eluted at a salt concentration comparable to that required to elute SarA (0.5 M). However, these studies were done in triplicate, and unlike SarA this protein was not consistently captured with any protease-associated bait in an amount sufficient to be visible by SDS-PAGE. Additionally, it was sometimes captured from lysates prepared from the LAC *sarA* mutant and in experiments done with LAC lysates using the SAU300_1445 control bait (data not shown). Nevertheless, in an attempt to identify this protein, as well as any additional proteins that may have been captured in amounts that were not visible by SDS-PAGE, we subjected eluates to LC-MS/MS analysis. Given the number of baits, the number of individual fractions obtained with each bait, and the need to carry out these experiments in triplicate to allow for robust statistical analysis, these studies were done using a 1:1 mixture of the 0.2 and 0.3 M NaCl eluates, the 0.5 M eluate alone, and a 1:1 mixture of the 0.75 and 1 M eluates from each triplicate sample. These experiments included a control without any DNA bait, thus allowing us to account for nonspecific binding to the streptavidin beads, and a control in which the DNA bait was derived from the ~400 bp region upstream of the gene encoding the segregation and condensation protein A (SAU300_1445), thus allowing us to distinguish proteins that bind to any functional promoter (e.g. those that constitute the basal transcription machinery) from those that bind specifically to each protease-associated promoter region. For statistical analysis, spectral counts obtained for each protein in each sample were summed and the resulting counts obtained with each bait compared to the spectral counts observed for the same protein in the control bait using a two-tailed t-test. Proteins with a p-value ≥ 0.05 were filtered out and the fold change (FC) was calculated for the remaining proteins. Proteins with an FC ≤ 2 were filtered out along with proteins with less than 8 spectral counts in at least 2 of 3 replicates; this cutoff was chosen based on counts obtained with the no DNA control samples.

Based on this analysis we concluded that the smaller protein eluted only at relatively high NaCl concentrations was most likely to be the nucleoid-associated protein HU. HU is an architectural protein involved in chromatin remodeling, but it is known to impact gene transcription [57]. Interestingly, it has also been suggested that SarA may serve a similar architectural role [58]. Thus, we cannot rule out the possibility that this protein impacts transcription of *S. aureus* genes including at least some that encode extracellular proteases, although it has never been shown to do so in any studies we are aware of. In any event, this protein did not meet our inclusion criteria for more detailed study, and we chose to focus on those proteins that met these criteria. To identify these, all proteins captured with each protease-related bait and the LC-MS/MS datasets obtained for each were compared with each other using Venny 2.1. The results confirmed that SarA was captured by all four baits as well as MgrA, Rot, and SarS (Fig. S1). With three of four baits, the protein with the highest number of spectral counts was SarA followed by MgrA, the exception being the *sspABC* bait in which the number of spectral counts observed with SarA and MgrA were comparable (Table S1). MgrA was previously shown to bind DNA baits associated with the *aur* and *sspABC* promoters [49], and a recent report demonstrated that mutation of *mgrA* limits transcription of *aur, sspABC*, and the *spl* operon but had little impact on transcription of *scpAB* [34]. This report was based on qRT-PCR analysis and did not examine MgrA binding specificity, but our results suggest that the regulatory impact of MgrA on the production of all extracellular proteases is likely to occur via a direct mechanism.

The other two proteins that were bound by all four baits are SarS and Rot. The number of spectral counts observed for SarS was generally greater than those observed for Rot with all four protease-associated baits, but even those observed for SarS were consistently lower than those observed for SarA (Table S1). Nevertheless, these findings are consistent with a previous study that found that Rot binds to all four protease promoters as assessed by electrophoretic mobility shift assays (EMSA) done with purified Rot and that mutation of *rot* could be correlated with increased overall protease activity and a reduced capacity to form a biofilm [59]. In contrast, previous reports that were limited to the *sspABC* and *aur* promoters found that SarS binds to the *sspABC* promoter but not the *aur* promoter [49,52]. Thus, to our knowledge this is the first report showing that SarS also binds the *aur, scpA*, and *spl* promoters. This suggests that, while both SarS and Rot are known to have global regulatory effects on the production of multiple proteins, they are likely to modulate the production of extracellular proteases directly rather than through an indirect mechanism involving other regulatory loci.

SarR and SarZ were bound by the promoter baits associated with *aur, sspABC*, and the *spl* operon, but based on our inclusion criteria neither was judged to specifically bind the bait associated with *scpAB* (Fig. S1). As with the impact of *sarA* on the *spl* operon, there are conflicting reports in the literature with respect to the impact of SarR in the protease regulatory circuit. For instance, there are reports concluding that SarR binds the *aur* and *sspABC* promoters and enhances transcription of both of these genes/operons [49,52], while another report concluded that mutation of *sarR* has no significant effect on transcription of the gene encoding aureolysin but does result in decreased transcription of the genes/operons encoding all other extracellular proteases [34]. SarR was first identified as a repressor of *sarA* transcription, and a *sarR* mutant was shown to produce increased levels of SarA [60]. Thus, it would be anticipated that mutation of *sarA* and *sarR* would have opposite effects on protease production, and transcriptional analysis by qRT-PCR indicate that this is generally the case [34]. However, to our knowledge, direct interactions between SarR and promoter regions associated with *scpA* and *splA-F* have not been previously reported. To the extent that the *scpA* promoter was the one exception with respect to SarR binding, these results also suggest that the impact of SarR on transcription of *aur, sspABC*, and the *spl* operon may occur via a direct mechanism, while the impact on *scpAB* transcription is likely to occur indirectly.

Similarly, SarZ has been implicated as a direct mediator of transcription of *sspABC* [37,51] but it has not been implicated in the direct transcriptional regulation of any of the other genes/operons encoding extracellular proteases. At the same time, mutation of *sarZ* has been correlated with altered transcription of a number of other genes known to impact protease production including *agr, sarA, sarS*, and *mgrA* [37]. In the recent report that examined the global network impacting protease production in *S. aureus*, mutation of *sarZ* was associated with increased protease activity, but it was not considered among the regulatory loci in the primary or secondary regulatory networks defined by this study, and its impact on transcription of individual genes/operons encoding extracellular proteases was not examined [34].

### *Relative impact of* S. aureus *regulatory proteins on protease production*

**Figure 2.**
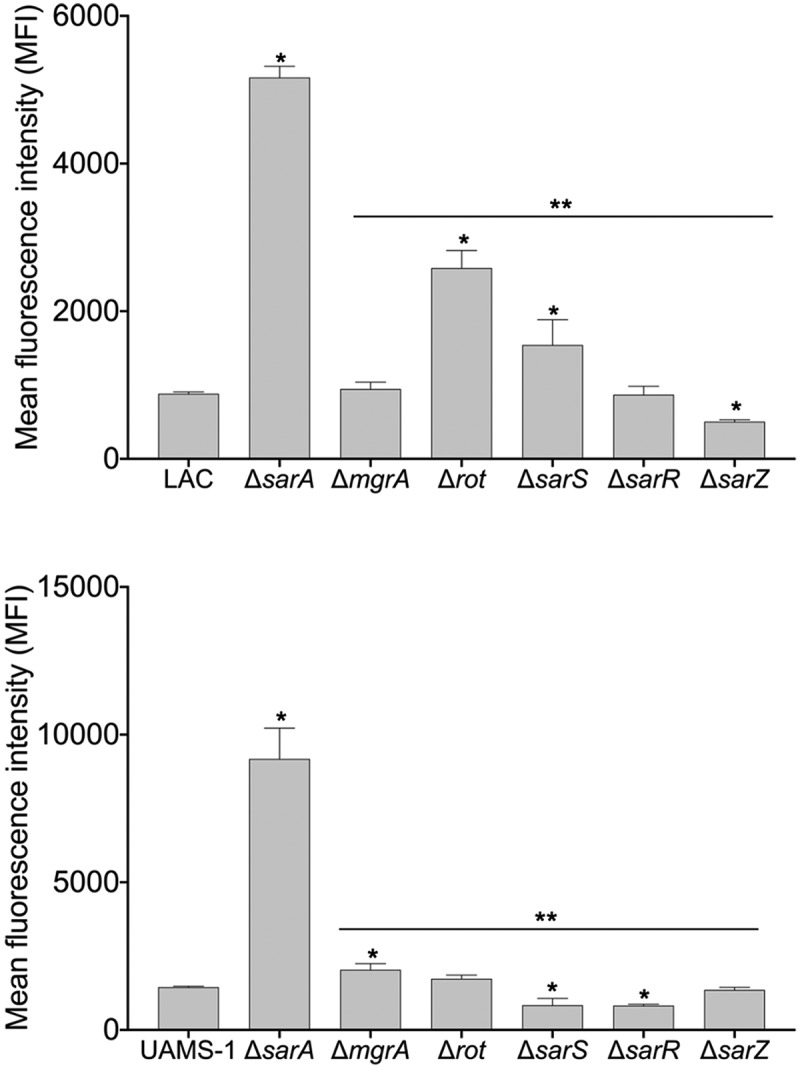
**Relative impact of regulatory mutations on protease activity**. Protease activity in conditioned medium (CM) from stationary phase cultures from LAC (top), UAMS-1(bottom), and each of the indicated isogenic regulatory mutants was assessed using a gelatin-based FRET assay. Results are reported as the average ± standard error of the mean from two biological replicates, each of which included three experimental replicates. Asterisk indicates statistical significance by comparison to the results observed with the isogenic parent strain. Doubles asterisks indicate statistical significance by comparison to the isogenic *sarA* mutant

These studies suggest that MgrA, Rot, SarA, SarR, SarS, and SarZ are likely to modulate the production of most if not all extracellular proteases directly, but they do not address the phenotypic impact of this binding. To address this, we mutated *mgrA, rot, sarA, sarR, sarS,* and *sarZ* in LAC and UAMS-1 and examined the impact on overall protease activity using a gelatin-based FRET assay. In LAC, mutation of *rot, sarA*, and *sarS* resulted in a statistically significant increase in protease activity, while mutation of *sarR* resulted in a slight but significant decrease (Figure 2). Overall, these results are consistent with a previous report demonstrating that mutation of *sarA, rot,* and *sarS* in the USA300 strain JE2 increased overall protease activity as assessed by gelatin zymography [34]

However, the results observed with UAMS-1 regulatory mutants differed by comparison to those observed with the corresponding LAC mutants. Specifically, mutation of *sarS* and *sarZ* resulted in a significant decrease in overall protease activity in UAMS-1, while mutation of *mgrA* resulted in a significant increase (Figure 2). In fact, only mutation of *sarA* resulted in a significant increase in protease activity in both LAC and UAMS-1. The increase observed in a UAMS-1 *mgrA* mutant is noteworthy in that qRT-PCR analysis found that mutation of *mgrA* in the USA300 strain Houston resulted in decreased expression of several genes/operons encoding extracellular proteases, most notably the *spl* operon [34].

**Figure 3.**
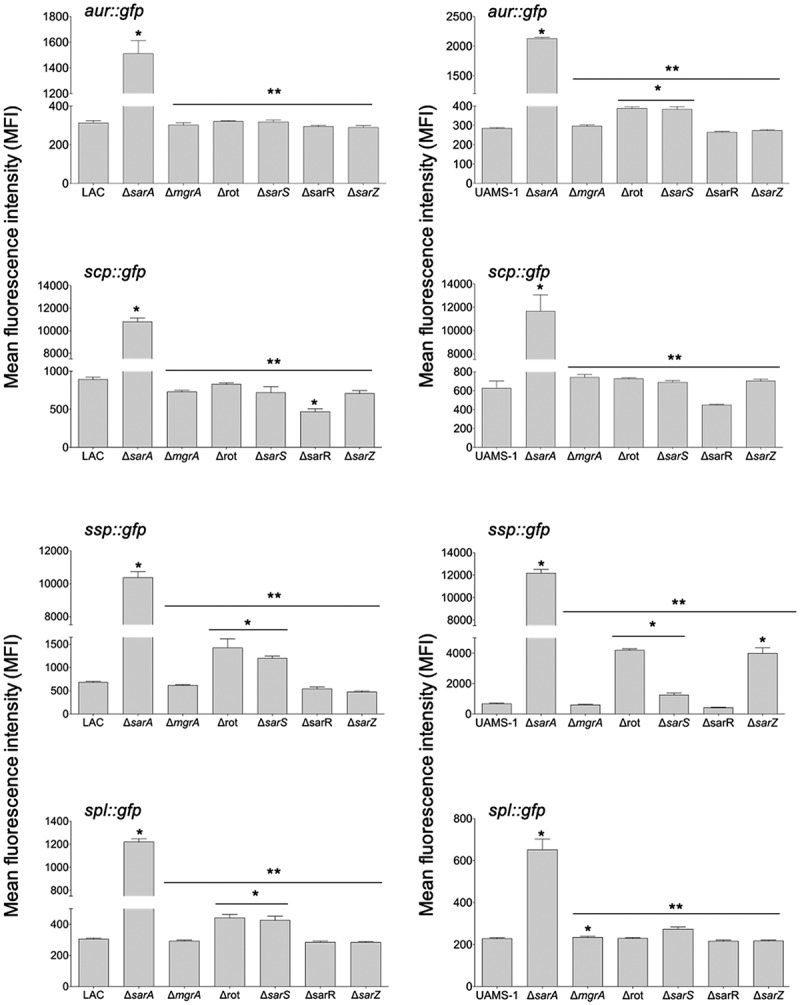
**Relative impact of regulatory loci on expression of individual protease genes and operons in LAC and UAMS-1**. The indicated pCM11 *gfp* reporter plasmids were introduced into LAC, UAMS-1 and each of the isogenic regulatory mutants. Results are reported as the average mean fluorescence intensity ± standard error of the mean from two biological replicates, each of which included three experimental replicates. Asterisk indicates statistical significance by comparison to the results observed with the isogenic parent strain. Doubles asterisks indicate statistical significance by comparison to the isogenic *sarA* mutant

To investigate this further, we employed pCM11-based transcriptional reporters constructed such that the same promoter regions used as DNA baits were used to drive expression of the *gfp* gene. These reporters were introduced into LAC, UAMS-1, and derivatives of each carrying mutations in each of the regulatory genes encoding proteins captured in our DNA bait assays. Fluorescence levels were significantly increased with all four reporters in *sarA* mutants generated in both LAC and UAMS-1 (Figure 3). In LAC, fluorescence was also significantly increased in *rot* and *sarS* mutants, but only with the *spl::gfp* and *ssp::gfp* reporters, and as with protease activity itself the increased fluorescence levels observed in LAC *rot* and *sarS* mutants with both of these reporters were well below those observed in the isogenic *sarA* mutants (Figure 3). These results indicate that in LAC the regulatory proteins Rot, SarA and SarS repress the production of at least a subset of extracellular proteases directly at a transcriptional level, but that SarA is the only regulatory protein that does so with all 10 extracellular proteases.

With the exception of the UAMS-1 *sarA* mutant, the results of our reporter assays with UAMS-1 regulatory mutants differed by comparison to the corresponding LAC mutants. The results were also less consistent with the results of assays assessing overall protease activity. Specifically, mutation of *rot* and *sarS* resulted in increased fluorescence with our pCM11 reporters with the *aur::gfp* and *ssp::gfp* reporters, but only mutation of *sarS* resulted in a significant increase in fluorescence with the *spl::gfp* reporter (Figure 3). Unlike the corresponding LAC mutant, a UAMS-1 *sarZ* mutant also exhibited increased fluorescence with the *ssp::gfp* reporter. However, in UAMS-1 only mutation of *mgrA* resulted in a significant increase in overall protease activity (Figure 2). The gelatin-based FRET assay we employed to assess overall protease activity does not reflect the production of all extracellular proteases, but it does readily detect aureolysin, SspA, and SspB, and in this respect, it is somewhat surprising that these changes were not reflected in our protease assays. However, as with the LAC mutants, all of the changes observed in our UAMS-1 reporter assays were minimal by comparison to the UAMS-1 *sarA* mutant (Figure 3). Thus, while the results of these reporter assays support the conclusion that multiple regulatory loci that bind protease-associated promoters impact transcription to a statistically significant extent, they also support the conclusion that SarA plays a predominant role in this regard. Indeed, as with protease activity itself (Figure 2), only mutation of *sarA* resulted in a significant increase in fluorescence with all reporters in both LAC and UAMS-1, and in both strains the increase observed in *sarA* mutants was significantly greater than that observed with the isogenic mutations generated in any of these other regulatory loci.

### *Impact of* S. aureus *regulatory proteins on the abundance of extracellular proteins*

The results discussed above do not preclude the possibility that mutation of regulatory loci other than *sarA* also impact *S. aureus* phenotypes of potential clinical relevance. More directly, the increased protease production observed in *sarA* mutants has been shown to play an important role in defining the reduced accumulation of many *S. aureus* proteins including alpha toxin and protein A, the reduced capacity to form a biofilm, and reduced virulence of *sarA* mutants in murine models of sepsis and osteomyelitis [27,28,56,61]. Thus, the question becomes whether the relatively limited impact of other regulatory mutations on protease production also significantly impacts these phenotypes.

**Figure 4.**
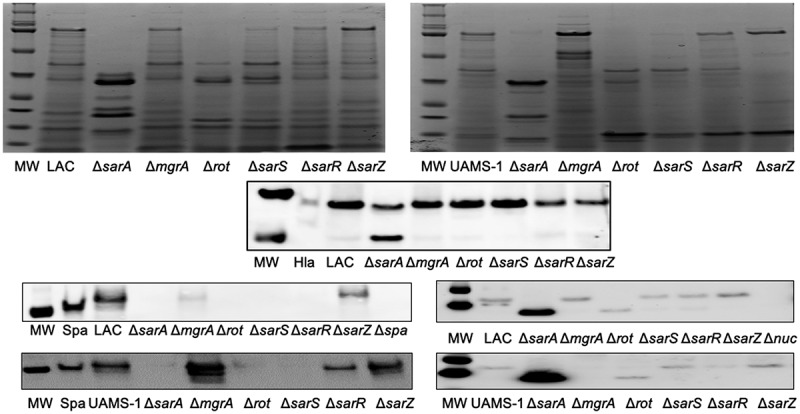
**Relative impact of regulatory loci on protein abundance. Top**: SDS-PAGE of conditioned medium (CM) from LAC (top), UAMS-1 (bottom) and isogenic regulatory mutants generated in each parent strain. **Bottom**: Western blots of CM from the same strains using antibodies for alpha toxin (Hla, top), protein A (Spa, left), and Nuc1 (right). Purified alpha toxin (Hla) was included as a control for alpha toxin blots (UAMS-1 does not produce alpha toxin), while protein A and *nuc1* mutants (Δ*spa* and Δ*nuc1*, respectively) were included as controls for the protein A and Nuc1 blots

To begin to address this, we first examined SDS-PAGE exoprotein profiles in the LAC and UAMS-1 regulatory mutants. Mutation of essentially all six loci had an impact on overall exoprotein profiles, which is not surprising given the global impact of all of these regulatory loci on protein production [30]. Differences in overall exoprotein patterns were apparent between LAC and UAMS-1 mutants, which is not surprising given known differences between these two strains. However, the overall impact of each mutation in both strains was virtually indistinguishable. Specifically, mutation of *sarA* resulted in a decrease in the abundance of full-length proteins in both strains that were visibly apparent by comparison to all of the other mutants (Figure 4). Mutation of *rot* and *sarS* also limited the accumulation of these proteins, albeit to a lesser extent by comparison to the isogenic *sarA* mutant. Notably, as assessed by SDS-PAGE, mutation of *mgrA* had little impact on the abundance of high molecular weight proteins in either strain despite the fact that it resulted in a slight but statistically significant increase in overall protease activity in UAMS-1 (Figure 2).

We also assessed the abundance of alpha toxin, protein A, and the Nuc1 extracellular nuclease by Western blot. These proteins were chosen based on our previous demonstration that all of these are impacted in LAC *sarA* mutants owing largely to protease-mediated degradation [27,28,62]. The abundance of alpha toxin was decreased to a greater extent in a LAC *sarA* mutant than any other regulatory mutant examined, and a smaller protein reactive with the anti-alpha toxin antibody was evident only in the LAC *sarA* mutant (Figure 4). Neither of these was the case with any other regulatory mutant, although mutation of *sarR* and *sarZ* in LAC did result in an apparent decrease in the abundance of full-length alpha toxin. In contrast, extracellular protein A was essentially absent in every mutant examined except the LAC *mgrA* and *sarZ* mutants (Figure 4). It was not possible to assess the abundance of alpha toxin in UAMS-1 because this strain does not produce alpha toxin. However, protein A was also absent in CM from every UAMS-1 mutant with the exception of the *mgrA, sarR*, and *sarZ* mutants. Indeed, the abundance of protein A was significantly increased in the UAMS-1 *mgrA* mutant by comparison to the isogenic parent strain despite the fact that mutation of *mgrA* was correlated with a slight increase in overall protease activity in UAMS-1 (Figure 2). Interestingly, the abundance of protein A was not increased in a LAC *mgrA* mutant despite the fact that no increase in protease activity was observed in this mutant.

The impact on Nuc1 varied both with respect to abundance and form. Specifically, Nuc1 is produced as a larger NucA form that is proteolytically cleaved to form a smaller NucB form. In both LAC and UAMS-1, only the NucB form was present in *sarA* and *rot* mutants (Figure 4), suggesting enhanced proteolytic cleavage. Additionally, the overall abundance of NucA was increased in LAC and UAMS-1 sarA mutants, and this was not the case with any other regulatory mutant examined.

### *Relative impact of* S. aureus *regulatory proteins on biofilm formation*

**Figure 5.**
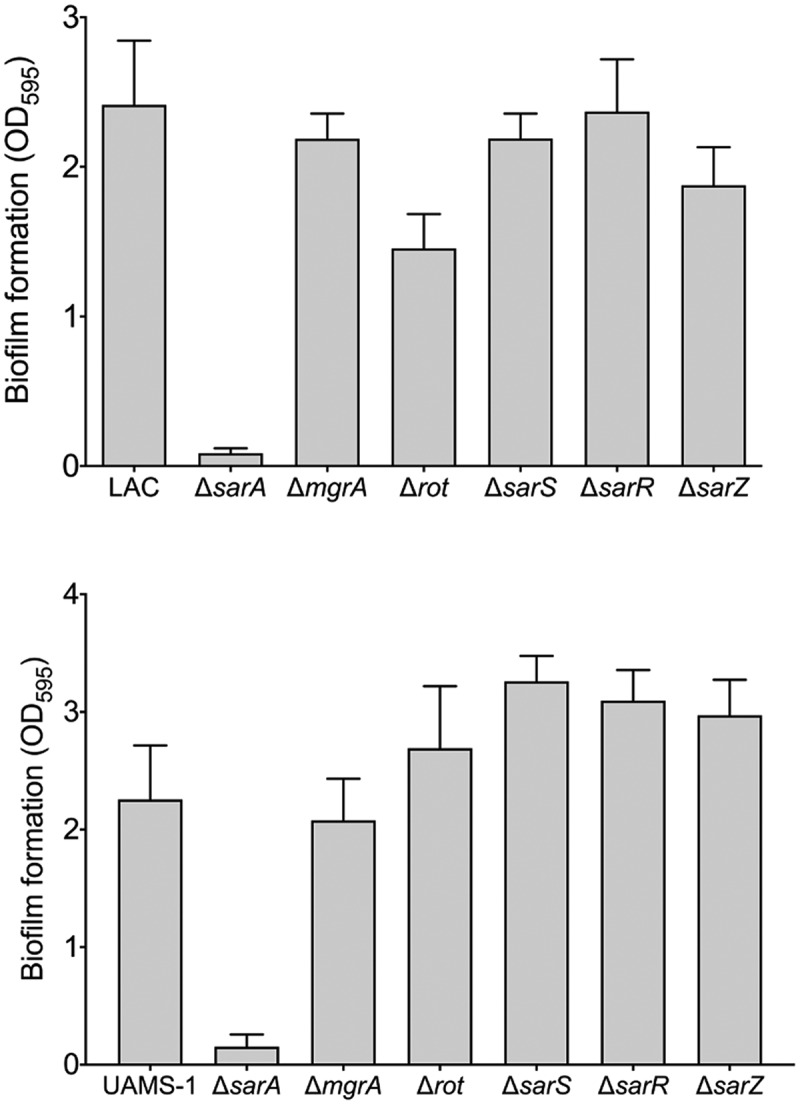
**Relative impact of regulatory loci on biofilm formation**. Biofilm formation was assessed in LAC (top), UAMS-1 (bottom) and their isogenic regulatory mutants. Results are reported as the average ± standard error of the mean from two biological replicates, each of which included six experimental replicates. Asterisk indicates statistical significance by comparison to the results observed with the isogenic parent strain. Doubles asterisks indicate statistical significance by comparison to the isogenic *sarA* mutant

Increased protease production has been shown to be a primary contributing factor to the reduced capacity of *S. aureus* regulatory mutants to form a biofilm [47,59,63–65]. Based on this we also examined the impact of mutations in the genes encoding each of the targeted proteins on biofilm formation. Just as mutation of *sarA* resulted in a greater increase in protease activity than any other mutation in both LAC and UAMS-1 (Figure 2), it also limited biofilm formation in both strains to a greater extent than any other regulatory mutation (Figure 5). In LAC, mutation of *rot* and *sarR* limited biofilm formation to a modest but statistically significant degree, while in UAMS-1 no other regulatory mutation was found to significantly limit biofilm formation. Indeed, mutation of *sarS, sarR*, and *sarZ* were all associated with a slight but statistically significant increase in biofilm formation in UAMS-1 (Figure 5). In contrast, mutation of *sarS* and *sarZ* had no significant impact on biofilm formation in LAC. Surprisingly, mutation of *sarR* was found to limit biofilm formation in LAC to a modest but statistically significant degree (Figure 5) despite the fact that it was also correlated with a decrease in overall protease activity (Figure 2).

### *Impact of the functional status of other regulatory loci on* sarA*-associated phenotypes*

**Figure 6.**
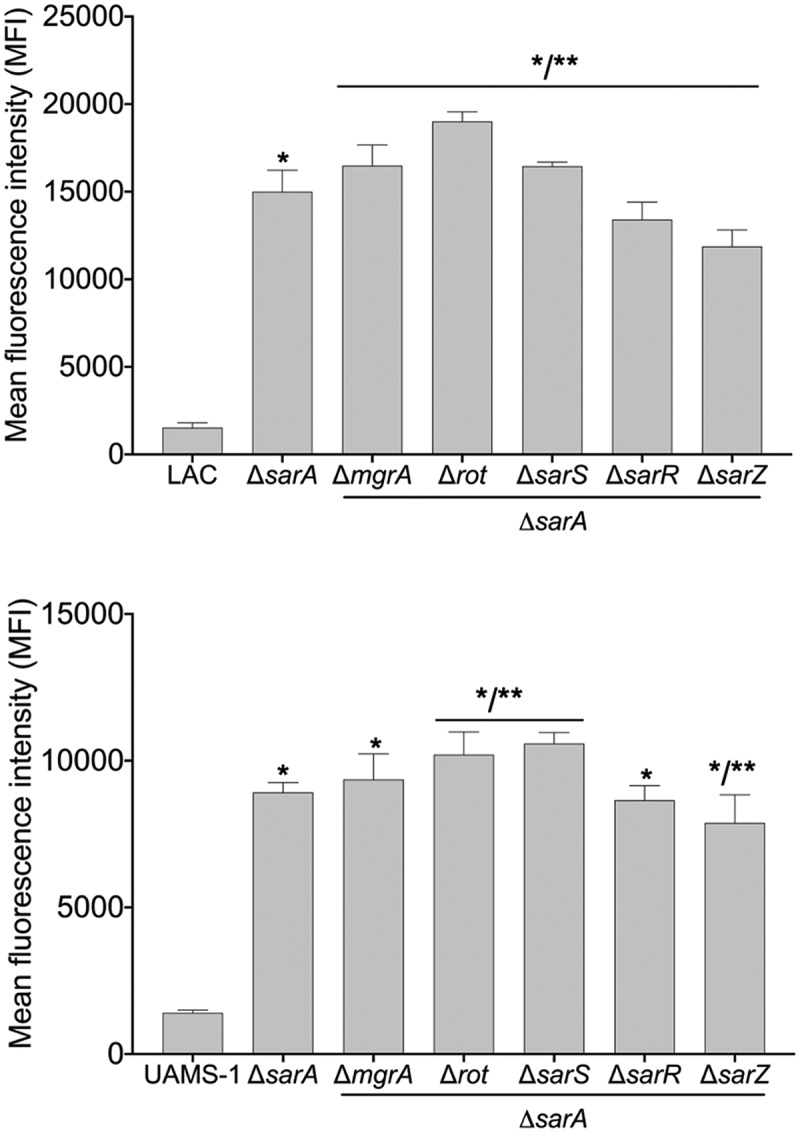
**Impact of the functional status of regulatory mutations on protease production in isogenic *sarA* mutants**. Protease activity in conditioned medium (CM) from stationary phase cultures from LAC, UAMS-1, and each of the indicated isogenic regulatory mutants was assessed using a gelatin-based FRET assay. Results are reported as the average ± standard error of the mean from two biological replicates, each of which included three experimental replicates. Asterisk indicates statistical significance by comparison to the results observed with the isogenic parent strain. Doubles asterisks indicate statistical significance by comparison to the isogenic *sarA* mutant

All of the results cited above support the conclusion that multiple regulatory loci bind protease-associated promoter regions and modulate protease production and protease-associated phenotypes. However, they also support the conclusion that none do so more than SarA in terms of their overall impact on these phenotypes or their impact in diverse clinical isolates of *S. aureus*. To investigate this further, we mutated each of the genes encoding regulatory proteins captured in our primary experiments in combination with *sarA* and examined these same phenotypes. As expected, protease production was significantly increased in both LAC and UAMS-1 *sarA* mutants, and mutation of each of the other regulatory loci had relatively little impact on protease production by comparison to the isogenic *sarA* mutant (Figure 6). That said, from a statistical perspective some differences were observed. Specifically, by comparison to the isogenic *sarA* mutant, protease production was increased in LAC *mgrA/sarA, rot/sarA*, and *sarS/sarA* mutants, while it was decreased in *sarR/sarA* and *sarZ/sarA* mutants. Protease production was also increased in UAMS-1 *rot/sarA* and *sarS/sarA* mutants by comparison to the *sarA* mutant, and slightly decreased in a UAMS-1 *sarZ/sarA* mutant (Figure 6).

**Figure 7.**
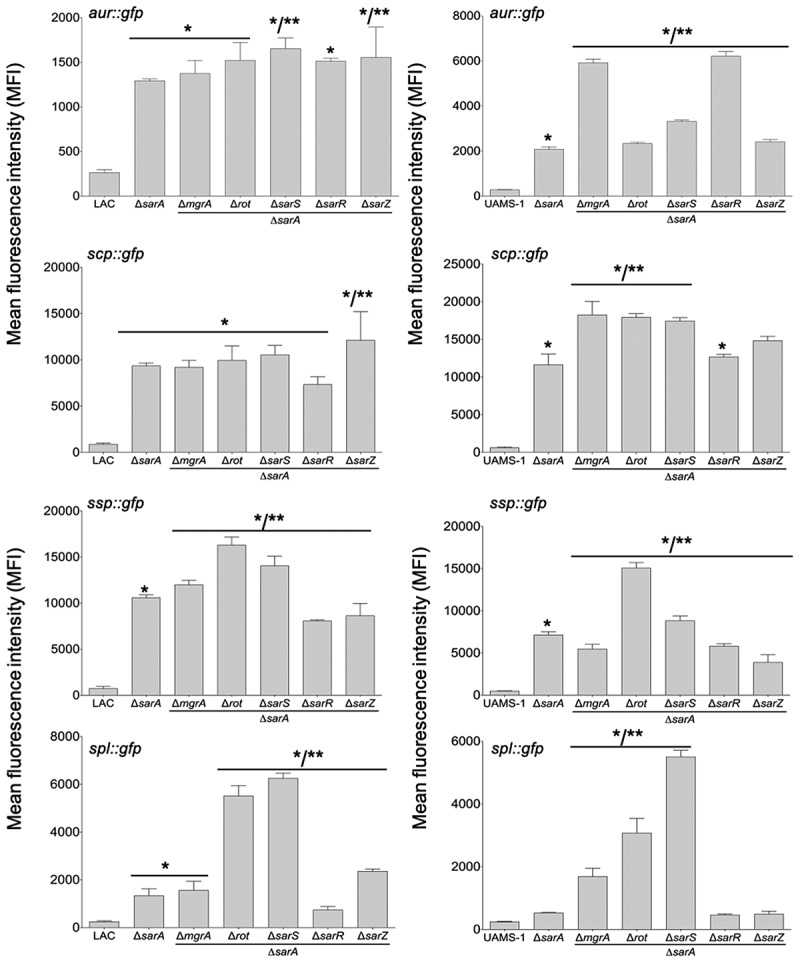
**Impact of the functional status of regulatory mutations on expression of protease genes/operons in LAC *sarA* mutants and UAMS-1 *sarA* mutants**. pCM11 *gfp* reporter plasmids were introduced into LAC or UAMS-1 and the indicated regulatory mutants. Results are reported as the average mean fluorescence intensity ± standard error of the mean from two biological replicates, each of which included three experimental replicates. Asterisk indicates statistical significance by comparison to the results observed with the isogenic parent strain. Doubles asterisks indicate statistical significance by comparison to the isogenic *sarA* mutant

Transcriptionally, at least as assessed using our pCM11 reporter assay, the results were clear, particularly in UAMS-1. For instance, in LAC fluorescence levels observed with the *aur::gfp, scp::gfp* and *ssp::gfp* reporters were comparable in *sarA* mutants and *sarA* mutants with concomitant mutations in other regulatory loci (Figure 7), and what differences were observed were generally consistent with the results of protease assays using the same strains (Figure 6). This was particularly true with *rot/sarA* and *sarS/sarA* mutants, both of which exhibited increased fluorescence with at least one of the reporters (Figure 7). This was also true with the *spl::gfp* reporter, and in fact the increase observed in these double mutants with this reporter was greater even by comparison to the isogenic *sarA* mutant. However, the *spl*-encoded proteases are not readily detectable in gelatin-based protease assays, so it is unlikely this contributed to the increased protease activity we observed in these mutants. In UAMS-1, fluorescence levels were increased relative to the isogenic *sarA* mutant in *rot/sarA* and *sarS/sarA* mutants with all four reporters (Figure 7). Interestingly, this was also true with the UAMS-1 *mgrA/sarA* mutant, while in LAC this was true only with the *ssp::gfp* reporter. Nevertheless, while we did observe some differences between strains, these results suggest that in both LAC and UAMS-1 maximal repression of protease production is dependent on multiple proteins, most notably SarA, Rot, and SarS.

**Figure 8.**
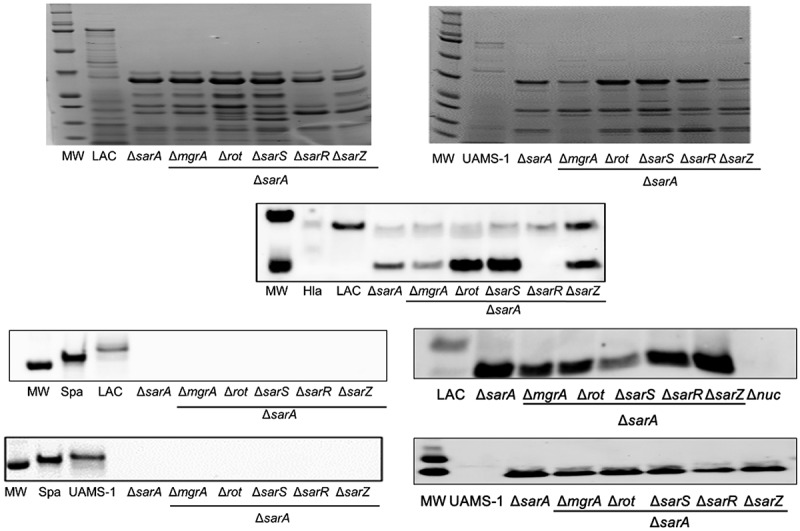
**Relative impact of regulatory loci on protein abundance. Top**: SDS-PAGE of conditioned medium (CM) from LAC (left), UAMS-1 (right), their isogenic *sarA* mutants, and isogenic *sarA* mutants with additional mutations in the genes encoding each of the other regulatory proteins examined in this study. **Bottom**: Western blots of CM from the same strains using antibodies for alpha toxin (Hla, top), protein A (Spa, left), and Nuc1 (right). Purified alpha toxin (Hla) was included as a control for alpha toxin blots (UAMS-1 does not produce alpha toxin), while protein A and *nuc1* mutants (Δ*spa* and Δ*nuc1*, respectively) were included as controls for the protein A and Nuc1 blots

**Figure 9.**
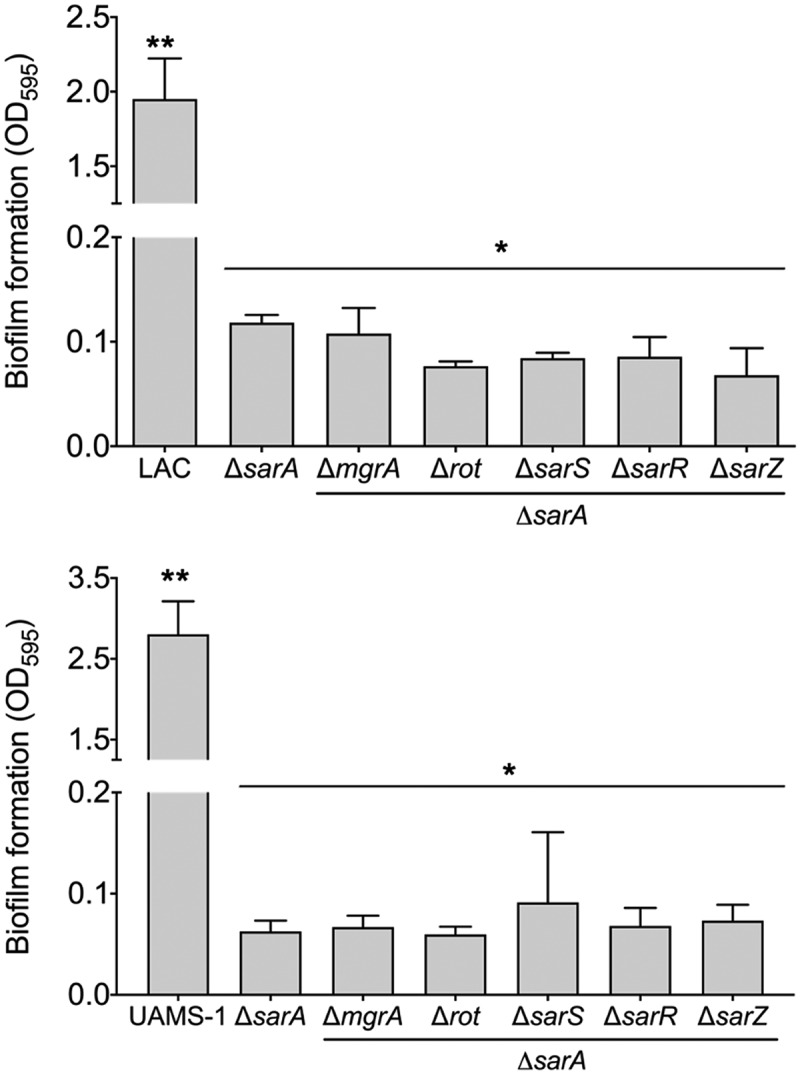
**Relative impact of regulatory loci on biofilm formation**. Biofilm formation was assessed in LAC (top), UAMS-1 (bottom), their isogenic *sarA* mutants, and isogenic *sarA* mutants with additional mutations in the genes encoding each of the other regulatory proteins examined in this study. Results are reported as the average ± standard error of the mean from two biological replicates, each of which included six experimental replicates. Asterisk indicates statistical significance by comparison to the results observed with the isogenic parent strain. Doubles asterisks indicate statistical significance by comparison to the isogenic *sarA* mutant

Such results illustrate the intricacy of *S. aureus* regulatory circuits that modulate protease production, but once again they do not address the phenotypic impact of the differences we observed in these mutants. Thus, we repeated our exoprotein analysis both by SDS-PAGE and by Western blot. In both LAC and UAMS-1, SDS-PAGE analysis confirmed that all of the double mutants examined exhibited a protein profile directly comparable to that observed in the isogenic *sarA* mutants (Figure 8). This was reflected in the decreased accumulation of full-length alpha toxin and/or increased accumulation of its degradation product in all LAC mutants other than the *sarR/sarA* mutant. It was also reflected in the absence of protein A in all mutants in both LAC and UAMS-1 (Figure 8). Additionally, only the smaller NucA form of Nuc1 was present in all *sarA* mutants irrespective of the functional status of any other regulatory gene (Figure 8). Biofilm formation was also reduced to an extent comparable to the isogenic *sarA* mutant in all other regulatory mutants examined (Figure 9).

### Impact of SarA on the binding of other regulatory proteins

**Figure 10.**
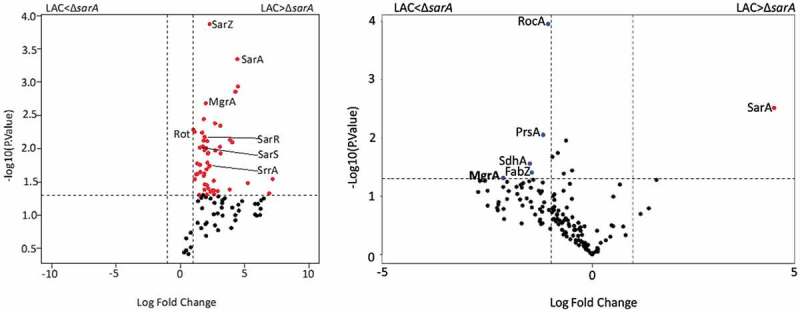
**Differential protein binding to the *spl* (left) and the aureolysin (right) promoters in LAC versus the isogenic Δ*sarA* mutant**. Volcano plots were generated based on fold-change of protein levels using the averaged spectral counts from biological triplicates. The x-axis indicates a log_2_ fold-change, and the y-axis indicates −log_10_ p-value based on the Student’s t-test. The horizontal line indicates a p-value <0.05, and the vertical lines represent a fold-change >2. Proteins in which the abundance was reduced to a statistically significant degree in Δ*sarA* lysates compared to wild-type lysates are shown as red dots in the upper right quadrant, whereas those that were present in increased amounts are shown as blue circles in the upper left quadrant. Black dots indicate proteins for which differences in abundance were not statistically significant

The results cited above demonstrate that multiple regulatory loci impact protease production and protease-associated phenotypes but that SarA plays a predominant role in this regard. They also suggest that all of these proteins impact protease production directly as evidenced by their identification using DNA capture assays. However, this does not preclude the possibility that their impact is mediated through an interaction with SarA. To begin to address this, we repeated our DNA bait assays using whole cell lysates prepared from a LAC *sarA* mutant. All five of the other proteins prioritized based on experiments done with LAC lysates were identified in these experiments as well, but their overall binding patterns were altered. For instance, none of the other five proteins bound all four baits (Fig. S2). Rot was captured with the *aur, splA-F*, and *sspAB*-associated baits, and SarZ bound the baits associated with *aur, scpAB*, and *sspABC*, while MgrA, SarR and SarS only bound the baits associated with *aur* and *sspABC*. Thus, the only two baits that bound the same proteins in experiments done with both LAC lysates and lysates prepared from its isogenic *sarA* mutant were those associated with *aur* and *sspABC* (Fig. S2). Irrespective of the bait used, the number of spectral counts observed with all proteins was also higher in experiments done with lysates prepared from LAC (Table S1) by comparison to those prepared from its isogenic *sarA* mutant (Table S2). This was most evident with the *spl*-associated bait in which the number of spectral counts observed with LAC lysates was higher than those observed with LAC Δ*sarA* lysates with all five proteins (Figure 10). Additional proteins were also identified in this comparison that bound to the spl-associated bait at higher levels in the presence of SarA, one example being the regulatory protein SrrA (Figure 10). One exception was MgrA and the aureolysin-associated bait. Specifically, the number of spectral counts observed in this context were higher with lysates prepared from the *sarA* mutant than those prepared from LAC itself (Table S2 and Figure 10). These results support the hypothesis that the impact of at least some regulatory loci on binding protease-associated promoters, and presumably protease production, may be dependent, at least in part, on the presence or absence of SarA.

### *Impact of individual proteases on* sarA*-associated phenotypes*

**Figure 11.**
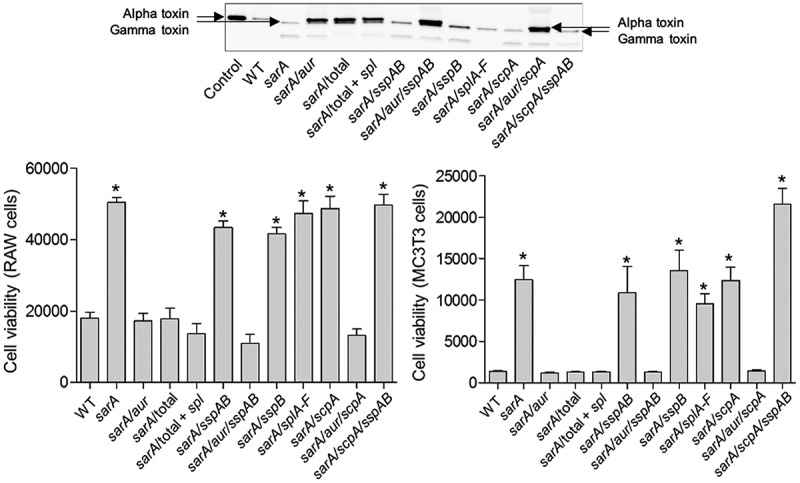
**Impact of individual proteases on accumulation of alpha toxin (top) and cytotoxicity (bottom). Top**: Conditioned medium (CM) from the indicated strains was resolved by SDS-PAGE prior to Western blotting with an anti-alpha toxin antibody. The control consists of purified alpha toxin. As previously described, this commercially available antibody is cross-reactive with gamma toxin.^13,60^ **Bottom**: Cytotoxicity was assessed using conditioned medium (CM) from stationary phase cultures of LAC (WT), its isogenic *sarA* mutant, and derivatives of the *sarA* mutant unable to produce the indicted proteases. Assays were done with RAW (left) or MC3T3 cells (right) as surrogates for primary osteoclasts and osteoblasts, respectively. Results are reported as relative viability. Single asterisk indicates a statistically significant increase in viability by comparison to CM from LAC (WT)

**Figure 12.**
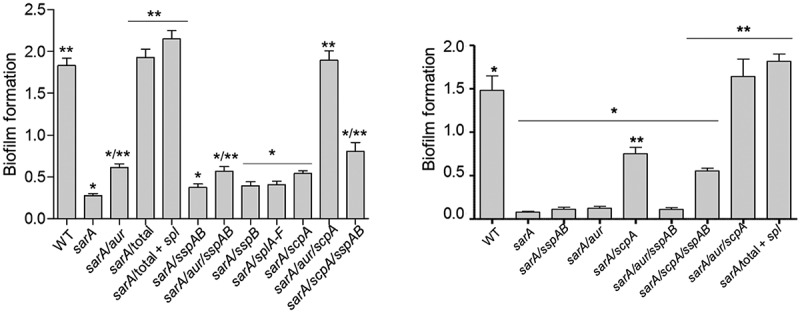
**Impact of individual specific proteases on biofilm formation**. Biofilm formation was assessed using a microtiter plate assay as previously described.^16^ WT refers to LAC (left) or UAMS-1 (right). “Total” refers to a *sarA* mutant unable to produce any extracellular protease. “Total + *spl*” refers to a *sarA* mutant unable to produce any extracellular protease other than those encoded by the *spl* operon. All other designations indicate the genes encoding specific proteases that were mutated in the LAC or UAMS-1 *sarA* mutants. Single asterisk indicates statistical significance relative to the isogenic parent strain. Double asterisks indicate statistical significance relative to the isogenic *sarA* mutant

To investigate the role of individual proteases in defining important phenotypes of *sarA* mutants, we generated mutations in the genes encoding individual proteases alone and in combination with each other in both LAC and UAMS-1 *sarA* mutants. The accumulation of full-length alpha toxin was essentially abolished in CM from a LAC *sarA* mutant, and it was restored to levels even beyond those observed in the LAC parent strain by eliminating the production of aureolysin (Figure 11). Since aureolysin sits at the top of an activation cascade that includes SspA and SspB, it is possible that this is due to the impact of eliminating aureolysin production on the activity of these proteases, but if that were the case it would be expected that the accumulation of alpha toxin would be restored by eliminating production of SspA and/or SspB, and this was not the case. Neither mutation of *scpA* nor the functional status of the *spl*-encoded proteases had any impact on the abundance of alpha toxin, thus suggesting that it is aureolysin itself that defines its reduced abundance in a LAC *sarA* mutant. Similarly, mutation of the gene encoding aureolysin was sufficient to restore the cytotoxicity of CM from a LAC *sarA* mutant for both osteoblasts and osteoclasts (Figure 11). In contrast, mutation of the gene encoding aureolysin alone was not sufficient to restore biofilm formation in LAC or UAMS-1 *sarA* mutants. Rather, in both strains this required concomitant mutation of the genes encoding both aureolysin and ScpA (Figure 12).

### Cis *elements involved in SarA-mediated repression of the expression of individual proteases*

We chose to focus on SarA in these experiments because it was the most abundant protein identified in our DNA bait assays and had a greater impact on protease production and protease-associated phenotypes than mutation of any of the other regulatory genes examined. Additionally, mutation of *sarA* had the most consistent impact on all of these phenotypes in both LAC and UAMS-1, and *sarA* mutants were the only mutants that exhibited increased expression of all four reporters in both strains. Thus, to gain insight into the molecular interaction between SarA and each protease-associated bait, we cloned sequentially truncated versions of the region upstream of each protease gene/operon starting with the same fragment used in our DNA bait assays and decreasing by 20–50 bp each time. These were cloned into our pCM11 *gfp* reporter [66] and introduced into LAC and its isogenic *sarA* mutant. Relative fluorescence levels were then assessed to determine whether it was possible to identify a specific region likely to contain one or more SarA binding sites. The rationale behind this approach was that it should be possible to replicate the phenotype of a *sarA* mutant even in the SarA-positive LAC parent strain in the absence of the relevant SarA binding site(s).

**Figure 13.**
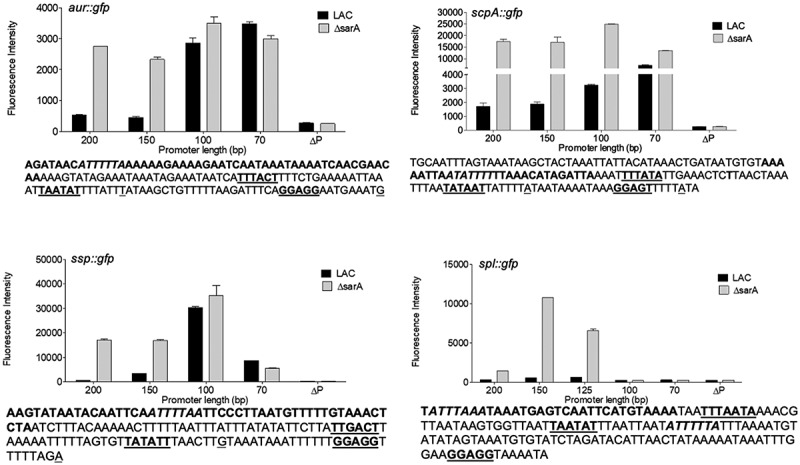
**Characterization of regions containing *cis* active elements that contribute to SarA-mediated repression of extracellular proteases**. Constructs containing the indicated number of base pairs upstream of each protease gene/operon translational start site were used to generate truncated versions of the pCM11 *gfp* reporter plasmid indicated in each panel. These were then introduced into LAC and its *sarA* mutant and fluorescence assessed after ON growth. Results are reported as the average mean fluorescence intensity ± standard error of the mean from two biological replicates, each of which included three experimental replicates. The sequence below each panel illustrates the region upstream of each protease gene/operon with the region containing putative SarA binding sites (bold italics), putative −35, −10, and ribosome-binding sites (bold underlined), and transcriptional and translational start sites (underlined)

As expected, increased fluorescence was observed in the LAC *sarA* mutant with all four full-length reporter constructs (Figure 13). The magnitude of the change did vary in a reporter-dependent manner, with the *scp* and *ssp* reporters exhibiting the greatest increase in fluorescence, which was perceptible even to the eye, followed by the *spl* reporter and lastly by the *aur* reporter. With the exception of the *spl::gfp* reporter (see below), this difference was apparent with every truncated construct generated with every reporter until the promoter region was reduced to a region containing 100 bp upstream of the translational start site of each protease-associated gene and/or operon (Figure 13). With the *aur* constructs, fluorescence was increased in LAC to a level comparable to that observed in the isogenic *sarA* mutant, and comparable to each other, with constructs containing 70 and 100 bp. This suggests the presence of one or more SarA binding sites between 100 and 150 bp upstream of the translational start site of the *aur* gene. Included within this region is a DNA site (ATTTTTA) comparable to the SarA binding site (ATTTTAT) defined by our previous SELEX experiments [67]. This putative SarA binding site is centered 69 bp upstream of the proposed −35 region [14] and 138 bp upstream of the translational start site. This is consistent with the results of our reporter assays and suggests that SarA-mediated repression of *aur* expression is unlikely to be due to competition for the RNA polymerase binding site.

The results observed with the *ssp::gfp* reporter were similar to those observed with the *aur::gfp* reporter in that fluorescence was increased to a comparable level in LAC and its *sarA* mutant with the construct extending 100 bp upstream of the *sspABC* translational start codon (Figure 13). This region also contains potential SarA binding sites (ATTTTAA or TTTTATT) as defined based on our previous SELEX analysis [67]. These overlapping sites are centered 68 or 69 bp upstream of the putative −35 box and 128 or 129 bp upstream of the translational start codon. By comparison to the 100 bp construct, fluorescence was significantly decreased in both LAC and its *sarA* mutant with the reporter construct containing 70 bp. There are several possible explanations, one being the loss of a binding site for an activator of protease production. Alternatively, while the location of the putative SarA binding site associated with the sspABC operon is located the same distance upstream of the proposed −35 site, it is possible the *sspABC* promoter itself may be compromised to some degree by deletion of the region between 70 and 100 bp. In this respect it is important to note that the key promoter elements have been proposed for the *sspABC* operon [14], but to our knowledge they have not been experimentally validated.

With the *scp::gfp* reporter, fluorescence was increased in LAC with the 100 bp construct, but to a lesser degree than was observed with the 70 bp construct (Figure 13). Additionally, with the 100 bp reporter construct, fluorescence was increased in both LAC and its isogenic *sarA* mutant. This suggests the existence of a binding site for a protein other than SarA in the region between 100 and 150 bp upstream of the translational start codon. Based on the results of our binding data indicating that SarZ binds the *scpAB* promoter region to a greater extent in the absence of SarA (Table S1 vs Table S2 and Fig. S1 vs Fig. S2), as well as reporter assays with double mutants (Figure 8), one candidate in this regard is SarZ. These results also suggest the absence of a SarA binding site in the 70 bp construct and, by extension, the presence of a SarA binding site in the region between 70 and 100 bp. This region upstream contains potential overlapping SarA binding sites (ATATTTT or ATTTTTT) centered 22 and 20 bp upstream of the putative −35 box and 85 or 83 bp upstream of the translational start codon [14].

The results observed with the *spl::gfp* reporter were different by comparison to all other reporters. Specifically, we observed essentially no fluorescence with a construct extending as far as 100 bp upstream of the translational start codon. To our knowledge, no promoter elements have been proposed for the *spl* operon, but based on those proposed for the other protease genes/operons the most likely candidates we could find were located further upstream (Figure 13), in which case this result would presumably reflect the absence of a functional promoter. Interestingly, there is a potential SarA binding site located between the putative ribosome-binding site and −10 box. Our assays do not allow us to assess the possible functionality of this site, but if it is a functional SarA binding site then it suggests that SarA binding may interfere with the binding of RNA polymerase to the *splA-F* promoter, thus suggesting a different regulatory mechanism by comparison to the other protease genes/operons examined. However, fluorescence was increased in a *sarA* mutant by comparison to LAC with constructs containing 125 and 150 bp of upstream DNA. This suggests the presence of a SarA binding site. Although no potential binding sites were identified in this region that matched the binding site defined by our SELEX experiments as well as the downstream site discussed above or any of the putative binding sites associated with each of the other promoter regions, there is a similar AT-rich region present in this location (Figure 13). Interestingly, fluorescence was decreased in both LAC and its *sarA* mutant with a 200 bp reporter construct by comparison to the constructs containing 150 and 125 bp, although it remained higher in the *sarA* mutant than in LAC. This suggests the presence of a binding site for an alternative repressor of *spl* expression in the region between 150 and 200 bp. Based on the results of our reporter assays using full-length reporter constructs (Figure 3), Rot and SarS would be potential candidates in this regard. This is consistent with the observation that expression of the full-length *spl::gfp* reporter was increased in *rot/sarA* and *sarS/sarA* mutants even by comparison to the isogenic *sarA* mutant (Figure 7).

All of these reporter assays were done with constructs generated with the promoter regions from LAC, but DNA sequence comparisons confirm that all of the putative regulatory elements, including the putative SarA binding site, are present and in the same location upstream of the UAMS-1 promoter regions associated with the genes/operons encoding aureolysin, SspA/SspB, and ScpA (data not shown). On the other hand, we did find differences in the promoter sequence upstream of the *spl* operon in LAC and UAMS-1. This was not surprising given that UAMS-1, as with EMRSA16, MRSA252, MN8, and several other *S. aureus* strains [10], does not encode SplA or SplB.

## Discussion

Changes in the production of extracellular proteases have been correlated with clinically relevant phenotypes of *S. aureus* including the accumulation of alpha toxin and protein A, biofilm formation, and virulence in diverse animal models [26–28,32,53,56,59,65,68,69]. Indeed, eliminating the production of aureolysin, SspA, SspB, ScpA, and the SplA-F proteases, or increasing the production of these same proteases, has been shown to have a dramatic impact on virulence and the overall *S. aureus* virulence factor repertoire [26–29]. Such observations underscore the importance of understanding the regulatory mechanisms that modulate protease production as a post-translational means of controlling the abundance of *S. aureus* virulence factors.

A growing number of regulatory loci have been implicated in the production of extracellular proteases. Indeed, one report found that mutation of >50 of 108 regulatory loci examined resulted in increased or decreased transcription of one or more of the genes encoding these proteases [34]. Similarly, a screen of the 1,920 mutants in the Nebraska Transposon Mutant Library (NTML) identified 62 mutants that exhibited differences in protease activity [70]. The experimental approach taken in the studies we report was based on two considerations. The first is that regulatory proteins that directly impact protease production would likely have a greater phenotypic impact than regulatory loci that impact protease production indirectly. The second is that proteins that regulate the production of all or most extracellular proteases are likely to have a greater impact on these phenotypes than regulatory proteins that have a less global impact.

Based on this, we used an unbiased chromatography approach using DNA baits derived from each of the four transcriptional units encoding the 10 primary *S. aureus* extracellular proteases (aureolysin, ScpA, SspA/SspB, and SplA-F). Based on SDS-PAGE, Western blot, and LC-MS/MS, we identified six *S. aureus* proteins (SarA, MgrA, SarS, Rot, SarR, and SarZ) that were bound by at least three of these four DNA baits. All of these have been previously implicated in protease production, in some cases to a degree that can be correlated with altered phenotypes of potential clinical relevance, most notably biofilm formation [27,28,34,59,71]. These studies confirmed that SarA is the most abundant protein captured with all four protease-associated baits followed by MgrA, SarS, and Rot in approximately that order. SarR and SarZ were captured by three of four baits, the exception in both cases being the ScpA-associated bait. However, it is important to note that SarR and SarZ were identified in LC-MS/MS analysis of proteins captured with the ScpA bait, but not to an extent that meet all of our inclusion criteria.

With few exceptions (e.g. Rot), it remains to be determined whether the capture of these proteins reflects a direct interaction with specific DNA targets or perhaps interactions among the proteins themselves. Nevertheless, subsequent studies analyzing derivatives of LAC and UAMS-1 with mutations in the six genes encoding each of these proteins confirmed that mutation of some of the genes encoding other regulatory proteins also resulted in increased protease production, decreased abundance of high molecular weight exoproteins, and decreased biofilm formation. The three that had the largest and most consistent impact were Rot, SarA and SarS, however, mutation of *sarA* had a significantly greater impact on all of these phenotypes by comparison to all other mutants including *rot* and *sarS* mutants. Moreover, *sarA* mutants were the only mutants that exhibited increased expression of all four protease genes/operons and did so in both LAC and UAMS-1. Additionally, this was also evident in the phenotype of derivatives of LAC and UAMS-1 with concomitant mutations in *sarA* and each of the other genes encoding regulatory proteins identified and prioritized based on the results of our binding assays. Specifically, mutation of *sarA* resulted in a greater increase in protease production, a greater decrease in the accumulation of high molecular weight proteins, and a more significant reduction in biofilm formation irrespective of the functional status of any of these other regulatory loci. Thus, we believe these collective results support the hypothesis that SarA plays a predominant role in limiting the production of *S. aureus* extracellular proteases.

However, this does not preclude an important role for other regulatory proteins in fine-tuning protease production. Indeed, in both LAC and UAMS-1 maximum expression of certain protease genes/operons was only observed when *sarA* was mutated along with other regulatory loci, the most notable among these once again being *rot* and *sarS*. This suggests that the functional status of *sarA* may influence the role of other regulatory proteins in modulating protease production. To examine this, we repeated our DNA bait assays using lysates prepared from a LAC *sarA* mutant. Interestingly, in the absence of SarA, all of the same regulatory proteins identified in our primary binding assays were found to bind protease-associated baits, but none were found to bind all four baits as defined by our statistical criteria. This reflects the fact that spectral counts observed with lysates from *sarA* mutants were generally reduced with all baits by comparison to those observed with lysates prepared from LAC. This was most apparent with the *spl*-associated bait in which MgrA, Rot, SarS, SarR and SarZ all bound to a significantly greater extent from lysates prepared from LAC than those prepared from its isogenic *sarA* mutant. However, there were also exceptions. Specifically, MgrA was bound by the *aur*-associated bait in greater abundance from the LAC *sarA* lysate than the lysate prepared from LAC itself. Mechanistically, one possible explanation for these findings is that MgrA competes with SarA for a similar or overlapping binding site, in which case the absence of SarA would presumably allow for increased binding. Alternatively, SarA could repress the production of MgrA, thus making it more abundant in a *sarA* mutant. Addressing these possibilities will require additional experiments that are beyond the scope of this report, but they nevertheless suggest that the functional status of *sarA* does impact the interaction between other regulatory proteins and specific protease promoter regions either directly at the protein-DNA level or indirectly through protein-protein interactions.

This impact of the functional status of *sarA* was also reflected in the results of our reporter assays in that we observed significant reporter-dependent differences with the double mutants. This made it important to determine whether specific proteases play a more important role than others in defining these potentially relevant *in vitro* phenotypes. We addressed this with an experimental focus on *sarA* for the reasons discussed above. Specifically, we generated *sarA* mutants that were unable to produce individual proteases alone and in combination with each other and examined the impact on the abundance of alpha toxin, cytotoxicity for osteoblasts and osteoclasts, and biofilm formation. The results confirmed that mutation of the gene encoding aureolysin alone was sufficient to restore the accumulation of alpha toxin in LAC as well as overall cytotoxicity for osteoblasts and osteoclasts. Similarly, mutation of the genes encoding aureolysin and ScpA was sufficient to fully restore biofilm formation in both LAC and UAMS-1 *sarA* mutants. This suggests that specific targets of these proteases may be particularly important. However, while mutation of *sarA* has been shown to attenuate virulence in murine models of sepsis and osteomyelitis owing to the increased production of extracellular proteases, these studies utilized *sarA* mutants unable to produce any extracellular protease [27,28,61], and it remains to be determined whether eliminating the production of these proteases alone or in combination with each other is sufficient to restore the virulence of *sarA* mutants in these same animal models. Additionally, using our reporter assays we did identify double mutants that exhibited increased expression of the *aur::gfp* and/or *scp::gfp* reporters even by comparison to the isogenic *sarA* mutant in both LAC and UAMS-1, and this raises the possibility that mutation of these regulatory loci may also attenuate virulence.

These results also raise the possibility that regulatory proteins that only bind specific protease-associated promoters are important. Indeed, we identified 11 proteins that bound only to the aureolysin promoter region (Table S3). Included among these was the redox-sensing transcriptional repressor (Rex), which has also been previously associated with protease production. Specifically, mutation of *rex* was shown to result in decreased transcription of *aur* as well as *sspABC* [34]. This suggests that Rex would not impact protease production in a manner that would limit the abundance of *S. aureus* virulence factors. Similarly, we identified six proteins that bound only to the *scpAB* promoter. Among these was a transcriptional regulator belonging to Xre family (SAUSA300_1969) that to our knowledge has not been functionally characterized. Interestingly, we identified 20 proteins that were captured only by the *splA-F* bait. Among these were the staphylococcal respiratory response protein A (SrrA), SarX, SarV, and a transcriptional regulator belonging to the MarR family encoded by SAUSA300_2452. Mutation of *sarV* has been shown to result in decreased transcription of the genes encoding aureolysin, ScpA and SplA [46], suggesting that SarV may function as a transcriptional activator. There is also at least one report indicating that *sarX* represses transcription of *ssp* [37]. Unlike the *aur, scpA*, and *spl*-associated baits, we did not identify any proteins that bound only the *ssp*-associated bait.

Finally, given the predominant role of SarA in repressing the production of all extracellular proteases, we carried out experiments using our reporter constructs that were aimed at identifying common DNA elements associated with SarA-mediated repression. Based on our earlier experiments that employed an unbiased SELEX (systematic evolution of ligands by exponential enrichment) approach to identify a functional SarA binding site [67], these studies allowed us to identify putative SarA binding sites upstream of the genes encoding aureolysin, ScpA, and SspA/SspB, but we were unable to do so in the region upstream of the *splA-F* operon. This is consistent with the observation that the results observed in all of our experiments focusing on the *spl* operon were generally distinct by comparison to those focusing on other protease genes/operons, and suggest that the mechanistic basis for the regulatory impact of SarA on expression of the *spl* operon may differ by comparison to its impact on expression of the genes encoding other proteases. Indeed, the *spl-*associated bait bound the highest number of proteins that did not bind any other protease-associated bait. Nevertheless, this does not change the observation that mutation of *sarA* results in increased expression of the *splA-F* operon and the increased accumulation of all six Spl proteases as previously reported [27,29]. Thus, while the results we report support the conclusion that multiple regulatory loci contribute to the control of *S. aureus* protease production, they also support the primary conclusion that *sarA* serves the predominant role in this regard. It is well known that *sarA* serves global regulatory roles, and we are not suggesting that other factors are not important, but we are confident that the results we report demonstrate that the impact of SarA on the production of extracellular proteases plays a key role in defining the phenotype of *S. aureus sarA* mutants.

## Materials and methods

### Bacterial strains and growth conditions

All bacterial strains used in this study are listed in Table 1. Those generated specifically for this study were generated by ɸ11-mediated transduction from previously described mutants in our culture collection [14,28,34,71–73] or available in the Nebraska Transposon Mutant Library (NTML). Primarily to generate the strains needed for our reporter assays, it was necessary to switch the resistance marker with the mutants in the NTML using previously described methods [74]. The exception was the UAMS-1 *sarS* mutant, which was generated using the pKOR1 mutagenesis system as previously described [75]. Strains were maintained at −80°C in tryptic soy broth (TSB) containing 25% (v/v) glycerol. For each experiment, strains were retrieved from cold storage by plating on tryptic soy agar (TSA) with appropriate antibiotic selection. Antibiotics were used at the following concentrations: chloramphenicol (Cm), 10 μg/mL; kanamycin (K), 50 μg/mL; neomycin (N), 50 μg/mL; erythromycin (Erm), 10 μg/mL; spectinomycin (Spec), 1 mg/mL; and tetracycline (Tet), 5 μg/mL. For phenotypic assays, 5 mL liquid cultures were grown overnight (ON) in TSB at 37°C with constant shaking without antibiotic selection.

**Table 1.** Bacterial strains used in this study

ID	Strain	Genotype	Reference or source
**Regulatory mutants**			
U-2279	LAC (USA300)	WT	[28]
U-2294	LAC (USA300)	*sarA*::K/N	[28]
U-4198	LAC (USA300)	*mgrA*::Cm	[76]
U-4309	LAC (USA300)	*mgrA:*:Cm/*sarA*::K/N	[64]
U-4291	LAC (USA300)	*rot*::Tet/	[64]
U-4726	LAC (USA300)	*rot*:Tet*/sarA*::K/N	This study.
U-4745	LAC (USA300)	*sarS*::Spec	[64]
U-4746	LAC (USA300)	*sarS*::Spec*/sarA*::K/N	This study.
U-4732	LAC (USA300)	*sarR*::Tet	This study.
U-4733	LAC (USA300)	*sarR*::Tet/*sarA*::K/N	This study.
U-4731	LAC (USA300)	*sarZ*::Spec	This study.
U-4734	LAC (USA300)	*sarZ*::Spec*/sarA::K/N*	This study.
U-1	U1 (USA200)	WT	[77]
U-929	U1 (USA200)	*sarA*::K/N	[72]
U-4185	U1 (USA200)	*mgrA*::Cm	[64]
U-4202	U1 (USA200)	*mgrA:*:Cm*/sarA*::K/N	This study.
U-4293	U1 (USA200)	*rot*::Tet	[64]
U-4727	U1 (USA200)	*rot*::Tet/*sarA*::K/N	This study.
U-1658	U1 (USA200)	*sarS*	This study.
U-1659	U1 (USA200)	*sarS*/*sarA*::K/N	This study.
U-4748	U1 (USA200)	*sarR*::Tet	This study.
U-4749	U1 (USA200)	*sarR*::Tet/*sarA*::K/N	This study.
U-4750	U1 (USA200)	*sarZ*::Tet	This study.
U-4751	U1 (USA200)	*sarZ*::Tet*/sarA::K/N*	This study.
**Protease deficient mutants**			
U-2296	LAC (USA300)	*aur*::Erm/*sarA*::K/N	This study.
U-3002	LAC (USA300)	*Δaur/Δssp/Δscp/spl*::Erm *sarA*::K/N	[28]
U-4218	LAC (USA300)	Δ*aur*/Δ*ssp*/Δ*scp*/*sarA*::K/N	[71]
U-4381	LAC (USA300)	Δ*ssp*/*sarA*::K/N	This study.
U-4382	LAC (USA300)	*Δaur*/Δ*ssp*/*sarA*::K/N	This study.
U-4397	LAC (USA300)	*sspB*::Erm*/sarA*::K/N	This study.
U-4398	LAC (USA300)	*spl*::Erm*/sarA*::K/N	This study.
U-4460	LAC (USA300)	*Δscp/sarA:*:K/N	This study.
U-4461	LAC (USA300)	Δ*aur*/Δ*scp*/*sarA*::K/N	This study.
U-4504	LAC (USA300)	*ssp*::Spec/*scp*::Tet/*sarA*::K/N	This study.
U-4120	U1 (USA200)	Δ*ssp*/*sarA*::K/N	This study.
U-4119	U1 (USA200)	Δ*aur*/*sarA*::K/N	This study.
U-4579	U1 (USA200)	*scpA*::Tet *sarA*::KN	This study.
U-4184	U1 (USA200)	Δaur/Δ*ssp*/*sarA*::K/N	This study.
U-4576	U1 (USA200)	Δ*ssp*/*scp*::Tet/*sarA*::K/N	This study.
U-4577	U1 (USA200)	Δ*aur*/*scp*::Tet/sarA::K/N	This study.
U-4578	U1 (USA200)	Δ*aur*/Δ*ssp*/*scp*::Tet/*sarA*::K/N	This study.

### DNA-affinity chromatography pull-down for isolation of proteins that bind protease-associated promoter regions

The DNA baits corresponding to each protease-associated promoter region were amplified by PCR. The oligonucleotides used for each amplification are listed in Table 2. In each case one of the oligonucleotides was biotinylated at the 5ʹ end. PCR fragments were purified using the Wizard® SV Gel and PCR Clean-Up System (Promega). Approximately 15 μg (calculated to saturate 50 μL of streptavidin beads (Miltenyi Biotec)) of DNA were incubated with 250 μL of whole cell lysates from *S. aureus* LAC or the isogenic Δ*sarA* mutant for 30 minutes at room temperature with constant shaking. The whole cell lysates were prepared using ON cultures standardized to and OD_560_ = 10. The cultures were centrifuged, the pellets were resuspended in BS/THES buffer [78] and transferred to Lysing Matrix B tubes (MP Biomedicals) the cells were lysed on FastPrep-24™ Classic Instrument. Streptavidin beads were added, and the mixture was immobilized in a μ Column (Miltenyi Biotec) in the magnetic field of the μMACS Separator (Miltenyi Biotec). Columns were previously equilibrated using the protein applications buffer (Miltenyi Biotec) and rinsed twice with 100 μL of BS/THES. After running the beads-DNA-lysates mixture through the column the unbound material was removed by washing 5 times with BS/THES. Bound proteins were eluted using buffers with increasing NaCl concentrations (0.1 M NaCl, 0.2 M NaCl, 0.3 M NaCl, 0.5 M NaCl, 0.75 M NaCl, and 1 M NaCl).

**Table 2.** List of oligonucleotides used to amplify the protease promoter elements

Gene	Forward primer	Reverse primer	Size (bp)
*aureolysin*	b-AGAGTTGTCGAAGTAACAGT	ATTTCATTCCTCCTGAAATCT	474
*scpA*	b-ATTGCATAGGTGTGGCATTT	TTGCAATAGGGGTAACACTT	425
*splA-F*	b-TTGCAATAGGGGTAACACTT	GGGGAACTAATCGCTAATAATGTG	461
*sspAB*	b-CCTCTCCTTTAAACACCTCA	TTGTCAAAGTTGCAACGAAT	464
SAU300_1445	b-ATAGTTGGCCTTGATGATGC	TAATATCTACCTCGTATTGCGT	467

### Identification of bound proteins

Each sample obtained with each protease-associated bait was visualized by SDS-PAGE and Western blot analysis with an α-SarA antibody, as previously described [28,29,71]. Additional analysis was done using liquid chromatography tandem mass spectrometry (LC-MS/MS). Specifically, samples were reduced, alkylated, and digested using filter-aided sample preparation [79] with sequencing grade-modified porcine trypsin (Promega). Tryptic peptides were then separated by reverse phase XSelect CSH C18 2.5 um resin (Waters) on an in-line 150 × 0.075 mm column using an UltiMate 3000 RSLCnano system (Thermo). Peptides were eluted using a 60 min gradient from 98:2 to 65:35 buffer A:B ratio (Buffer A = 0.1% formic acid, 0.5% acetonitrile; Buffer B = 0.1% formic acid, 99.9% acetonitrile). Eluted peptides were ionized by electrospray (2.2 kV) followed by mass spectrometric analysis on an Orbitrap Fusion Lumos mass spectrometer (Thermo). MS data were acquired using the FTMS analyzer in profile mode at a resolution of 240,000 over a range of 375 to 1500 m/z. Following HCD activation, MS/MS data were acquired using the ion trap analyzer in centroid mode and normal mass range with precursor mass-dependent normalized collision energy between 28.0 and 31.0. The analysis was done in triplicate for each DNA bait using a 1:1 mixture of the 0.2 M and 0.3 M NaCl eluates, the 0.5 M eluate, and a 1:1 mixture of the 0.75 M and 1 M NaCl eluates.

### Data analysis

Proteins were identified by database search using Mascot (Matrix Science, version 2.5.1) against the *USA300 S. aureus* database (2653 entries, Genebank accession JTJK01000002). A decoy database (based on the reverse of the protein sequences) was used in the search to calculate the FDR for the search algorithm. The search parameters include fixed modification of carbamidomethyl on cysteine, variable modifications of acetylation on the protein N-terminus, variable modification of oxidation on methionine, a maximum of two missed cleavages with trypsin, parent ion tolerance of 3 ppm, and fragment ion tolerance of 0.5 Da. Scaffold (Proteome Software) was used to verify MS/MS-based peptide and protein identifications. Peptide identifications were accepted if they could be established with less than 1.0% false discovery by the Scaffold Local FDR algorithm. Protein identifications were accepted if they could be established with less than 1.0% false discovery and contained at least 2 identified peptides. Protein probabilities were assigned by the Protein Prophet algorithm [80]. Total spectral counts for each replicate were exported from Scaffold into Microsoft Excel for further analysis.

The spectral counts obtained for each identified protein from each of the three eluates obtained with each sample were summed and the resulting counts obtained with each protease promoter compared to the spectral counts found for the same protein present in the samples obtained using the promoter element of the control gene SAUSA300_1445 using two-tailed t-test. Proteins with a p-value ≥ 0.05 were filtered out and the fold change was calculated for the remaining proteins. Proteins with an FC ≤ 2 were filtered out along with proteins with less than 8 spectral counts in at least two out of the three replicates (this number was chosen based on counts obtained from the no DNA control samples). The lists of proteins meeting these criteria were compared and visualized with a Venn diagram created using Venny (version 2.1). The Venn diagrams represent the number of shared and unique proteins identified binding to each promoter element. Volcano plots, generated using R studio, were used to visualize the significant changes in proteins binding to each bait depending on the presence/absence of SarA. The *y*-axis consists of −log_10_ *p*-values based on Student’s *t* test analysis, and the *x*-axis consists of the log_2_ fold change.

### Extracellular protease activity

These assays were done as previously described [27]. Briefly, 5 mL overnight cultures grown without antibiotic selection. To account for slight variations in optical density, all cultures were standardized relative to each other based on an optical density (OD_560_) of 10.0) and filter sterilized to obtain CM. Protease activity was assessed using the EnzChek Gelatinase/Collagenase Assay Kit (Thermo) according to the manufacturer’s instructions. The final gelatin fluorescein conjugate substrate concentration used in each assay was 25 μg/mL. Fluorescence intensity was read after a 23 h incubation period using the FLUOstar Omega Multi-Mode Microplate Reader (BMG LABTECH) (excitation filter = 485 nm emission filter = 520 nm.

### Transcriptional reporter assays

For each protease gene/operon amplicons ranging from 150 to 500 bp immediately upstream of the translational start site were cloned using Gibson Assembly Cloning (SGI-DNA) into pCM11 between the HindIII and KpnI sites. Additional constructs containing shorter promoter elements (50–125) were constructed by amplifying the regions of interest by PCR using primers including the HindIII and KpnI restriction sites and ligating into pCM11. The promoterless control plasmid (ΔP) was constructed using the QuikChange II XL Site-Directed Mutagenesis Kit to delete the region between the HindIII and KpnI sites on pCM11. All primers used to generate the inserts for these constructs are listed in Table 3. These plasmids were constructed and cloned in *E. coli* One Shot TOP10 (Thermo) and subsequently transformed into *S. aureus* strain RN4220 by electroporation [81] before transduction into appropriate *S. aureus* strains using phage ɸ11. Fluorescence was assessed as previously described [56]. Briefly, ON cultures were standardized by optical density. For single regulatory mutants all assays were done after standardizing conditioned medium (CM) to an OD_560_ of 10. For mutants containing more than one regulatory mutation, assays were done with CM standardized to an OD_560_ of seven because some of these mutants did not reach an OD_560_ of 10 given the high levels of GFP being expressed. 200 µL of the standardized cultures were plated into black clear-bottomed 96-well plates and the fluorescence intensity was measured with a FLUOstar Omega microplate reader (excitation, 485 nm; emission, 520 nm) (BMG Labtech).

**Table 3.** List of plasmids and oligonucleotides used in this study

Plasmid	Oligonuecleotide	IS*
pCM11::*aur*450-*gfp*	Fwd:GTTGTTAAGCTT-**CATACACCATACAAAACAAG**	450
pCM11::*aur*200-*gfp*	Fwd:GTTGTTAAGCTT-**TAAATTTTAAAATATAATTAAGC**	200
pCM11::*aur*150-*gfp*	Fwd: GTTGTTAAGCTT–**TAAATTTTAAAATATAATTAAGC**	150
pCM11::*aur*100-*gfp*	Fwd: GTTGTTGTTAAGCTT-**AAAGTATAGAAATAAATAGAA**	100
pCM11::*aur*70-*gfp*	Fwd: GTTGTTAAGCTT-**AAAAGTATAGAAATAAATAGAAATAAT**	70
pCM11::*aur-gfp*	Rev:GTTGTTGGTACC-**CTTATAAATAAAATATTAATTTAATTTTTC**	
pCM11::*scpA*500-*gfp*	Fwd:GTTGTTAAGCTT-**TACTTCTCCTATTGTATGG**	500
pCM11::*scpA*200-*gfp*	Fwd:GTTGTTAAGCTT-**TAATATATTTTCATGAAACTTTCG**	200
pCM11::*scpA*150-*gfp*	Fwd:GTTGTTAAGCTT-**TTGCAATTTAGTAAATAAGC**	150
pCM11::*scpA*100-*gfp*	Fwd: GTTGTTGTTAAGCTT-**AAAAATTAATTATTTTTTAAAC**	100
pCM11::*scpA*70-*gfp*	Fwd: GTTGTTGTTAAGCTT-**AAATTTTATATTGAAACTC**	70
pCM11::*scpA-gfp*	Rev: GTTGTTGTTACC-**TATAAAAACTCCTTTATTTTATTATAAAAT**	
pCM11::*splA*500-*gfp*	Fwd:GTTGTTAAGCTT-**CACTTAATCTGATCTCCGAAATAAC**	500
pCM11::*splA*200-*gfp*	Fwd: GTTGTTGTTAAGCTT-**AAATAATCCTAAAAAGTCAATTTT**	200
pCM11::*splA*150-*gfp*	Fwd:GTTGTTGTTAAGCTT-**TATTTAAATAAATGAGTCAATTCAT**	150
pCM11::*splA*125-*gfp*	Fwd: GTTGTTGTTAAGCTT-**GTAAAATAATTTAATAAAACG**	125
pCM11::*splA*100-gfp	Fwd:GTTGTTGTTAAGCTT-**GTTAATTAATATTTAATT**	100
pCM11::*splA*70-*gfp*	Fwd:GTTGTTGTTAAGCTT**T-AAAATGTATATAGTAAATGTG**	70
pCM11::*splA-gfp*	Rev: GTTGTTGTTACC-**TATTTTACCTCCTTCCAAATT**	
pCM11::*sspA*500-*gfp*	Fwd:GTTGTTAAGCTT-**AACTAAATTCATAGTACGTTCAG**	200
pCM11::*sspA*200-*gfp*	Fwd: GTTGTTGTTACC-**TAGATAAATGCAAACAATTGACG**	200
pCM11::*sspA*150-*gfp*	Fwd:-GTTGTTGTTACC-**AAGTATAATACAATTCAAT**	150
pCM11::*sspA*100-*gfp*	Fwd: GTTGTTGTTAAGCTT**-TCTTTACAAAAACTTTTTAAT**	100
pCM11::*sspA*70-*gfp*	Fwd: GTTGTTGTTAAGCTT-**TATTCTTATTGACTTAAA**	70
pCM11::*sspA-gfp*	Rev: GTTGTTGTTACC-**TCTAAAAACCTCCAAAAAATT**	
pCM11ΔPromoter	Fwd: AACGACGGCCAGTGCCACCTTAGGAGGATG	0
pCM11ΔPromoter	Rev: CATCCTCCTAAGGTGGCACTGGCCGTCGTT	0

*IS: Insert size in base pairs

### Exoprotein profiles and Western blotting

CM collected as described above was resolved by SDS-PAGE using 4% to 12% gradient Novex Bis-Tris Plus gels (Life Technologies). Proteins were visualized by staining with SimplyBlue SafeStain (Life Technologies). Images were obtained using a Bio-Rad ChemiDoc MP imaging system (Bio-Rad Laboratories). The accumulation of alpha-toxin (Hla), protein A (Spa), and the NucA and NucB forms of Nuc1 were assessed in these samples by Western blot as previously described [28,29,71].

### Biofilm formation

Biofilms were assessed as previously described [82]. Briefly, 5 mL ON cultures grown in biofilm media (TSB supplemented with dextrose and NaCl) were standardized by optical density OD_560_ of 0.05 and inoculated into non-tissue culture treated 96-well plates previously coated with 20% human plasma and incubated at 37°C. Biofilms were stained with crystal violet before eluting with ethanol and measuring the absorbance at 595 nm with a FLUOstar Omega microplate reader.

### Cytotoxicity

Cell viability was assessed on confluent monolayers of MC3T3-E1 and RAW 264.7 cells (10,000 and 50,000 cells/well, respectively) after 24 hour incubation with a 1:1 mixture of CM from standardized *S. aureus* stationary cultures and mammalian cell growth media, as previously described [61]. Cytotoxicity was determined using the LIVE/DEAD Viability/Cytotoxicity Kit for mammalian cells (Thermo Fisher Scientific) according to the manufacturer’s instructions.

### Statistical analysis

All assays were done in triplicate with at least two independent biological replicates. Statistical analyses were performed using GraphPad Prism 5.0 (GraphPad Software, La Jolla, CA). Statistical significance was determined by one-way ANOVA with Dunnett’s correction. Separate comparisons were made with all strains relative to LAC or to its Δ*sarA* mutant. A *p*-value ≤0.05 was considered statistically significant.

## Supplementary Material

Supplemental Material
