# Supplementary material for: SarA plays a predominant role in controlling the production of extracellular proteases in the diverse clinical isolates of Staphylococcus aureus LAC and UAMS-1
Source: Virulence. 2020 Dec 14;11(1):1738–62. doi: 10.1080/21505594.2020.1855923 (PMC7738309; doi:10.1080/21505594.2020.1855923)
Supplement: Supplemental Material [file KVIR_A_1855923_SM8104.pdf]

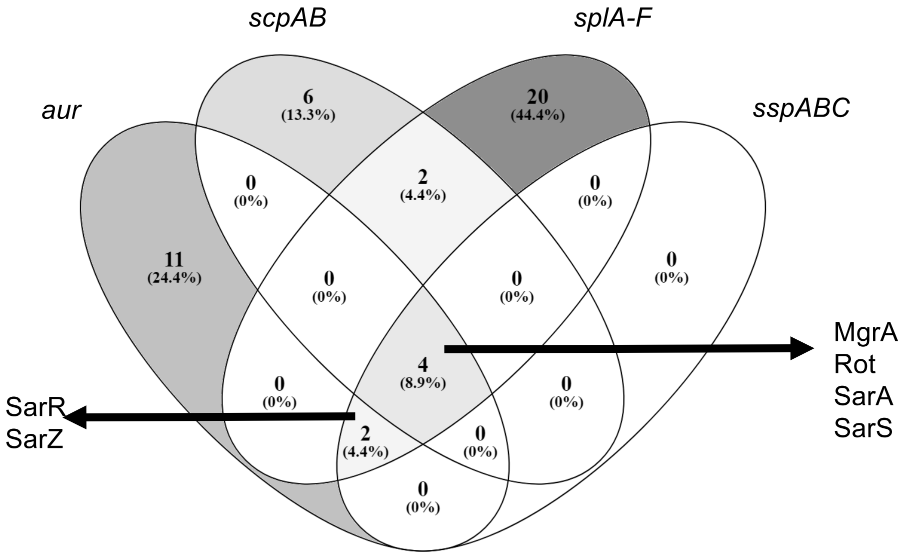

Supplementary Fig. 1. **Venn diagram indicating overlap between proteins present in the DNA-affinity chromatography pull-down with protease promoter-associated DNA.** Whole cell lysates from three independent stationary phase cultures of LAC were incubated with DNA corresponding to upstream regions (~400 bp) from the translational start sites of the genes/operons encoding aureolysin (*aur*), SplA-F (*splA-F*), ScpA (*scpAB)* and SspA/SspB (*sspABC*). Samples were subsequently loaded onto a streptavidin column and proteins bound to the DNA bait eluted from the column using a buffer containing increasing concentrations of salt (0.2-1M). The eluates were collected and analyzed by LC/MS/MS. The number of proteins identified as significantly differing ($p \leq 0.05$; $\log_2$ FC ≥ 2) between the protease-related DNA and a non-protease associated promoter control are shown.

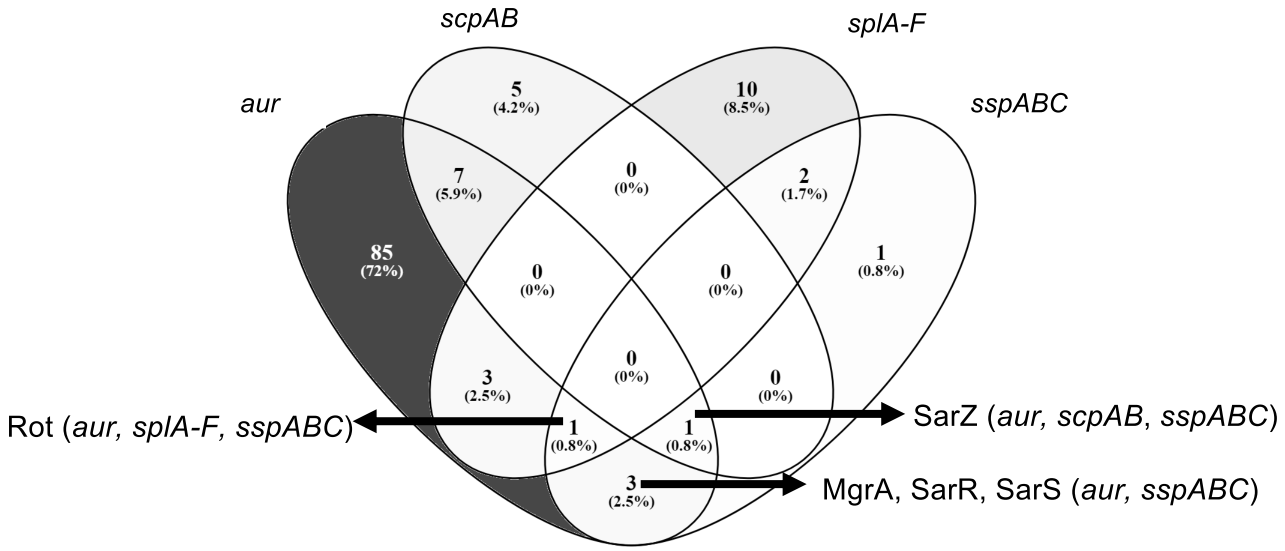

Supplementary Fig. 2. **Venn diagram indicating overlap between proteins present in the DNA-affinity chromatography pull-down with protease promoter-associated DNA.** Whole cell lysates from three independent stationary phase cultures of a LAC *sarA* mutant were incubated with DNA corresponding to upstream regions (~400 bp) from the translational start sites of the genes/operons encoding aureolysin (*aur*), SplA-F (*splA-F*), ScpA (*scpAB)* and SspA/SspB (*sspABC*). Samples were subsequently loaded onto a streptavidin column and proteins bound to the DNA bait eluted from the column using a buffer containing increasing concentrations of salt (0.2-1M). The eluates were collected and analyzed by LC/MS/MS. The number of proteins identified as significantly differing ($p \leq 0.05$; $\log_2$ FC ≥ 2) between the protease-related DNA and a non-protease associated promoter control are shown.

Protein	DNA bait:				
	aur	*scpAB*	*splA-F*	*sspABC*	SAU300_1445
SarA	212-338 (261)	547-636 (595.3)	160-217 (194.3)	97-283 (218.3)	27-36 (31.3)
MgrA	73-84 (79)	64-191 (125)	67-94 (84)	63-224 (222.5)	14-23 (20)
SarS	38-55 (45.7)	41-75 (60)	43-68 (57)	26-43 (37)	14-14 (12.7)
Rot	14-19 (16)	14-16 (14.7)	18-25 (22.3)	8-13 (10.7)	1-2 (1.7)
SarR	24-35 (30.3)	5-17 (12)	13-20 (16)	19-26 (22.7)	1-4 (0.3)
SarZ	16-24 (19.3)	9-12 (10.5)	21-25 (22.7)	22-42 (35.3)	2-8 (5)

Supplementary Table 1. **Spectral counts observed with LAC whole cell lysates.** Numbers indicate the range of spectral counts observed with triplicate samples for each individual protein and each individual DNA bait. Number in parenthesis indicates the average for each combination. SAU300_1445 is the control bait derived from a DNA region that is not associated with any protease gene or operon.

Protein	DNA bait: Range (average)				
	aur	*scpAB*	*splA-F*	*sspABC*	SAU300_1445
MgrA	157-172 (166.7)	45-108 (66.7)	17-24 (21.3)	63-173 (126.3)	14-23 (20)
SarS	40-60 (49)	25-60 (41)	16-26 (19.7)	27-55 (44)	14-14 (12.7)
Rot	20-28 (23.33)	1-2 (1.6)	10-11 (10.3)	12-20 (15.7)	1-2 (1.7)
SarR	16-25 (19)	7-15 (11)	4-8 (5.7)	12-22 (16.7)	1-4 (0.3)
SarZ	22 (22)	12-19 (16)	2-8 (5)	34-111 (78.7)	2-8 (5)

Supplementary table 2. **Spectral counts observed with whole cell lysates prepared from a LAC Δ*sarA* mutant.** Numbers indicate the range of spectral counts observed with triplicate samples for each individual protein and each individual DNA bait. Number in parenthesis indicates the average for each combination. SAU300_1445 is the control bait derived from a DNA region that is not associated with any protease gene or operon.

Supplementary Table 3.

Identified Proteins Binding Aureolysin Bait	Accession Number	Gene	ASC* (LAC)	ASC* (Δ*sarA*)
Transcriptional regulator SarA OS=Staphylococcus aureus (strain USA300) GN=sarA PE=3 SV=1	Q2FJ20	*sarA*	261.0	12.0
Transcriptional regulator, MarR family OS=Staphylococcus aureus (strain USA300) GN=SAUSA300_0672 PE=4 SV=1	A0A0H2XDE4	*mgrA*	79.0	166.7
Staphylococcal accessory regulator OS=Staphylococcus aureus (strain USA300) GN=SAUSA300_0114 PE=4 SV=1	A0A0H2XHA2	*sarS*	45.7	49.0
HTH-type transcriptional regulator SarR OS=Staphylococcus aureus (strain USA300) GN=sarR PE=3 SV=3	Q2FEJ8	*sarR*	30.3	19.0
Penicillin-binding protein 2 OS=Staphylococcus aureus (strain USA300) GN=mecA PE=4 SV=1	A0A0H2XHY4	*mecA*	23.0	43.7
Redox-sensing transcriptional repressor Rex OS=Staphylococcus aureus (strain USA300) GN=rex PE=3 SV=1	Q2FF78	*rex*	22.3	15.7
Serine hydroxymethyltransferase OS=Staphylococcus aureus (strain USA300) GN=glyA PE=3 SV=1	Q2FF15	*glyA*	21.3	24.3
HTH-type transcriptional regulator SarZ OS=Staphylococcus aureus (strain USA300) GN=sarZ PE=3 SV=1	Q2FEB2	*sarZ*	19.3	22.0
30S ribosomal protein S13 OS=Staphylococcus aureus (strain USA300) GN=rpsM PE=3 SV=1	Q2FER3	*rpsM*	19.0	21.0
Putative universal stress protein SAUSA300_1656 OS=Staphylococcus aureus (strain USA300) GN=SAUSA300_1656 PE=3 SV=1	Q2FG28	*SAUSA300_1656*	18.7	20.0
Carbamoyl-phosphate synthase large chain OS=Staphylococcus aureus (strain USA300) GN=carB PE=3 SV=1	Q2FHN5	*carB*	18.0	30.0
Pyruvate oxidase OS=Staphylococcus aureus (strain USA300) GN=cidC PE=3 SV=1	A0A0H2XGE9	*cidC*	17.0	19.7
HTH-type transcriptional regulator rot OS=Staphylococcus aureus (strain USA300) GN=rot PE=3 SV=1	A0A0H2XFX7	*rot*	16.0	23.3
ATP synthase subunit beta OS=Staphylococcus aureus (strain USA300) GN=atpD PE=3 SV=1	Q2FF24	*atpD*	16.0	33.3
Putative phosphoesterase SAUSA300_0916 OS=Staphylococcus aureus (strain USA300) GN=SAUSA300_0916 PE=3 SV=1	Q2FI62	*SAUSA300_0916*	15.7	27.7
Putative lipoprotein OS=Staphylococcus aureus (strain USA300) GN=SAUSA300_0693 PE=4 SV=1	A0A0H2XH45	*SAUSA300_0693*	15.0	20.3
Amino acid ABC transporter, amino acid-binding protein OS=Staphylococcus aureus (strain USA300) GN=SAUSA300_2359 PE=4 SV=1	A0A0H2XK42	*SAUSA300_2359*	14.0	19.3

Identified Proteins Binding ScpAB Bait	Accession Number	Gene	ASC* (LAC)	ASC*

				**(Δ*sarA*)**
Transcriptional regulator SarA OS=Staphylococcus aureus (strain USA300) GN=sarA PE=3 SV=1	Q2FJ20	*sarA*	595.3	5
DNA-directed RNA polymerase subunit beta' OS=Staphylococcus aureus (strain USA300) GN=rpoC PE=3 SV=2	Q2FJ97	*rpoC*	374.7	158.0
DNA-directed RNA polymerase subunit beta OS=Staphylococcus aureus (strain USA300) GN=rpoB PE=3 SV=2	Q2FJ98	*rpoB*	286.3	286.3
Phi77 ORF011-like protein, phage transcriptional repressor OS=Staphylococcus aureus (strain USA300) GN=SAUSA300_1969 PE=4 SV=1	A0A0H2XG68	*SAUSA300_1969*	157.0	53.0
Transcriptional regulator, MarR family OS=Staphylococcus aureus (strain USA300) GN=SAUSA300_0672 PE=4 SV=1	A0A0H2XDE4	*mgrA*	125.3	66.7
3-hydroxyacyl-[acyl-carrier-protein] dehydratase FabZ OS=Staphylococcus aureus (strain USA300) GN=fabZ PE=3 SV=1	Q2FF28	*fabZ*	120.7	142.7
Staphylococcal accessory regulator OS=Staphylococcus aureus (strain USA300) GN=SAUSA300_0114 PE=4 SV=1	A0A0H2XHA2	*sarS*	60.0	41.0
RNA polymerase sigma factor SigA OS=Staphylococcus aureus (strain USA300) GN=rpoD PE=3 SV=1	A0A0H2XKA5	*rpoD*	39.3	14.7
DNA-binding protein HU OS=Staphylococcus aureus (strain USA300) GN=hup PE=3 SV=1	A0A0H2XF50	*hu*	23.3	59.7
50S ribosomal protein L21 OS=Staphylococcus aureus (strain USA300) GN=rplU PE=3 SV=1	Q2FG80	*rplU*	16.7	10.3
HTH-type transcriptional regulator rot OS=Staphylococcus aureus (strain USA300) GN=rot PE=3 SV=1	A0A0H2XFX7	*rot*	14.7	14.3
Probable DNA-directed RNA polymerase subunit delta OS=Staphylococcus aureus (strain USA300) GN=rpoE PE=3 SV=1	Q2FF00	*rpoE*	14.0	3.0

**Identified Proteins Binding SplA-F Bait**	**Accession Number**	**Gene**	**ASC* (LAC)**	**ASC* (Δ*sarA*)**
Transcriptional regulator SarA OS=Staphylococcus aureus (strain USA300) GN=sarA PE=3 SV=1	Q2FJ20	*sarA*	194.3	9.0
5'-nucleotidase, lipoprotein e(P4) family OS=Staphylococcus aureus (strain USA300) GN=SAUSA300_0307 PE=4 SV=1	A0A0H2XKC1	*SAUSA300_0307*	111.3	5.0
Aldehyde dehydrogenase OS=Staphylococcus aureus (strain USA300) GN=aldA2 PE=3 SV=1	A0A0H2XFU7	*aldA2*	93.7	22.0
Transcriptional regulator, MarR family OS=Staphylococcus aureus (strain USA300) GN=SAUSA300_0672 PE=4 SV=1	A0A0H2XDE4	*mgrA*	84.0	21.3
Staphylococcal accessory regulator OS=Staphylococcus aureus (strain USA300) GN=SAUSA300_0114 PE=4 SV=1	A0A0H2XHA2	*sarS*	57.0	19.7

DNA-directed RNA polymerase subunit beta' OS=Staphylococcus aureus (strain USA300) GN=rpoC PE=3 SV=2	Q2FJ97	*rpoC*	49.0	5.0
Phosphoenolpyruvate carboxykinase [ATP] OS=Staphylococcus aureus (strain USA300) GN=pckA PE=3 SV=1	Q2FFV5	*pckA*	42.0	6.3
1-pyrroline-5-carboxylate dehydrogenase OS=Staphylococcus aureus (strain USA300) GN=rocA PE=3 SV=1	Q2FDV3	*rocA*	41.7	15.0
Staphylococcal respiratory response protein, SrrA OS=Staphylococcus aureus (strain USA300) GN=srrA PE=4 SV=1	A0A0H2XI68	*srrA*	34.7	7.7
2,3-bisphosphoglycerate-dependent phosphoglycerate mutase OS=Staphylococcus aureus (strain USA300) GN=gpmA PE=3 SV=1	Q2FE81	*gpmA*	32.3	6.0
DNA-directed RNA polymerase subunit beta OS=Staphylococcus aureus (strain USA300) GN=rpoB PE=3 SV=2	Q2FJ98	*rpoB*	31.7	5.0
Putative 2-hydroxyacid dehydrogenase SAUSA300_2254 OS=Staphylococcus aureus (strain USA300) GN=SAUSA300_2254 PE=3 SV=1	Q2FEI9	*SAUSA300_2254*	24.3	5.0
HTH-type transcriptional regulator SarZ OS=Staphylococcus aureus (strain USA300) GN=sarZ PE=3 SV=1	Q2FEB2	*sarZ*	22.7	4.7
HTH-type transcriptional regulator rot OS=Staphylococcus aureus (strain USA300) GN=rot PE=3 SV=1	A0A0H2XFX7	*rot*	22.3	10.3
Transaldolase OS=Staphylococcus aureus (strain USA300) GN=SAUSA300_1725 PE=3 SV=1	A0A0H2XET4	*SAUSA300_1725*	21.3	5.7
Molecular chaperone Hsp31 and glyoxalase 3 OS=Staphylococcus aureus (strain USA300) GN=hchA PE=3 SV=1	Q2FJ89	*hchA*	16.7	3.7
HTH-type transcriptional regulator SarR OS=Staphylococcus aureus (strain USA300) GN=sarR PE=3 SV=3	Q2FEJ8	*sarR*	16.0	4.3
Bacterial non-heme ferritin OS=Staphylococcus aureus (strain USA300) GN=ftnA PE=3 SV=1	Q2FFK2	*ftnA*	13.3	4.7
UPF0337 protein SAUSA300_0816 OS=Staphylococcus aureus (strain USA300) GN=SAUSA300_0816 PE=3 SV=1	Q2FIG2	*SAUSA300_0816*	13.0	0.7
Uncharacterized protein OS=Staphylococcus aureus (strain USA300) GN=SAUSA300_0814 PE=4 SV=1	A0A0H2XGY2	*SAUSA300_0814*	12.0	1.3
Purine nucleoside phosphorylase DeoD-type OS=Staphylococcus aureus (strain USA300) GN=deoD PE=3 SV=1	A0A0H2XEF6	*deoD*	11.7	1.3
Formimidoylglutamase OS=Staphylococcus aureus (strain USA300) GN=hutG PE=3 SV=1	Q2FEG2	*hutG*	11.3	1.3
DNA topoisomerase 1 OS=Staphylococcus aureus (strain USA300) GN=topA PE=3 SV=2	Q2FHI8	*topA*	10.3	0.2
Staphylococcal accessory regulator OS=Staphylococcus aureus (strain USA300) GN=SAUSA300_2218 PE=4 SV=1	A0A0H2XJC2	*sarV*	9.7	2.7
Catabolite control protein A OS=Staphylococcus aureus (strain USA300) GN=ccpA PE=4 SV=1	A0A0H2XH43	*ccpA*	9.7	0.7

HTH-type transcriptional regulator SarX OS=Staphylococcus aureus (strain USA300) GN=sarX PE=3 SV=2	Q2FIX1	*sarX*	9.3	3.7
Transcriptional regulator, MarR family OS=Staphylococcus aureus (strain USA300) GN=SAUSA300_2452 PE=4 SV=1	A0A0H2XFK7	*SAUSA300_2452*	8.7	1.3
ATP-dependent Clp protease ATP-binding subunit ClpC OS=Staphylococcus aureus (strain USA300) GN=clpC PE=3 SV=1	Q2FJB5	*clpC*	8.3	2.0

**Identified Proteins Binding SspABC Bait**	**Accession Number**	**Gene**	**ASC* (LAC)**	**ASC* (Δ*sarA*)**
Transcriptional regulator, MarR family OS=Staphylococcus aureus (strain USA300) GN=SAUSA300_0672 PE=4 SV=1	A0A0H2XDE4	*mgrA*	222.5	126.3
Transcriptional regulator SarA OS=Staphylococcus aureus (strain USA300) GN=sarA PE=3 SV=1	Q2FJ20	*sarA*	218.3	2.0
Staphylococcal accessory regulator OS=Staphylococcus aureus (strain USA300) GN=SAUSA300_0114 PE=4 SV=1	A0A0H2XHA2	*sarS*	37.0	44.0
HTH-type transcriptional regulator SarZ OS=Staphylococcus aureus (strain USA300) GN=sarZ PE=3 SV=1	Q2FEB2	*sarZ*	35.3	78.7
HTH-type transcriptional regulator SarR OS=Staphylococcus aureus (strain USA300) GN=sarR PE=3 SV=3	Q2FEJ8	*sarR*	22.7	16.7
HTH-type transcriptional regulator rot OS=Staphylococcus aureus (strain USA300) GN=rot PE=3 SV=1	A0A0H2XFX7	*rot*	10.7	15.7

*ASC=Spectral Counts Average
